# Molecular phylogeny of *Anopheles nivipes* based on mtDNA-COII and mosquito diversity in Cambodia-Laos border

**DOI:** 10.1186/s12936-022-04121-w

**Published:** 2022-03-17

**Authors:** Yilong Zhang, Canglin Zhang, Rui Yang, Chunhai Luo, Yan Deng, Yan Liu, Yilong Zhang, Hongning Zhou, Dongmei Zhang

**Affiliations:** 1 Department of Tropical Diseases, Faculty of Naval Medicine, Naval Medical University, Shanghai, 200433 China; 2 Yunnan Institute of Parasitic Diseases, Yunnan Provincial Key Laboratory of Vector-Borne Diseases Control and Research, Yunnan Provincial Center of Malaria Research, Yunnan Provincial Collaborative Innovation Center for Public Health and Disease Prevention and Control, Yunnan Institute of Parasitic Diseases Innovative Team of Key Techniques for Vector Borne Disease Control and Prevention (Developing), Pu’er, 665099 China

**Keywords:** Malaria, Mosquitoes, COII, Molecular phylogeny

## Abstract

**Background:**

Few studies have been conducted to investigate the distribution of mosquito vectors and the population structure of secondary vectors in the border region of Cambodia-Laos. The aim of this work was to study the mosquito diversity and molecular phylogeny of secondary vectors, i.e., *Anopheles nivipes* in this area.

**Methods:**

1440 adult mosquitoes were trapped in the Cambodia-Laos border. mtDNA-COII were amplified and sequenced from 53 *An. nivipes* DNA samples. Together with COII sequences deposited in GenBank, a total of 86 COII sequences were used for examining population variations, genetic differentiation, spatial population structure, population expansion, and gene flow patterns.

**Results:**

The adult mosquitoes were classified into 5 genera and 27 species in this border region. The predominant genera were *Culex* (60.07%, 865/1440) and *Anopheles* (31.25%, 450/1440), and the major *Anopheles* species were *An. nivipes* (73.56%, 331/450) and *Anopheles maculatus* (14.22%, 64/450). Based on sequences analysis of COII, a high level of genetic differentiation was reported in two Northwest India (Cheema and Bathinda, Punjab) and Cambodia-Laos (Siem Pang, Stung treng) populations (*F*_ST_ = 0.97824, 0.97343, *P* < 0.05), as well as lower gene flow (Nm = 0.01112, 0.01365) in the *An. nivipes* populations. Phylogenetic analysis and SAMOVA revealed a gene barrier restricting gene flow among three *An. nivipes* populations. Mantel test suggested a significant correlation between geography and gene distance in all *An. nivipes* populations (Z = 44,983.1865, r = 0.5575, *P* = 0.0070). Neutrality test and Mismatch distribution revealed a recent population expansion of *An. nivipes* in the Cambodia-Laos population.

**Conclusions:**

*Anopheles nivipes* was one of the major *Anopheles* species in the Cambodia-Laos border. Based on sequences analysis of COII, a genetic barrier between Cambodia-Laos and two Indian populations was found, and a recent population expanding or selecting of *An. nivipes* occurred in the Cambodia-Laos population, suggesting that COII might be an effective marker for describing the molecular phylogeny of *An. nivipes.* Further investigation and continuous surveillance of *An. nivipes* are warranted in this region.

**Supplementary Information:**

The online version contains supplementary material available at 10.1186/s12936-022-04121-w.

## Background

Dengue fever and malaria continue to be critical parasitic diseases jeopardizing people’s lives in subtropical and tropical regions, primarily in the Greater Mekong Subregion (GMS, Myanmar, Vietnam, Laos, China, Thailand, and Cambodia) [1]. Over the past 15 years, Cambodia has effectively controlled malaria through the introduction of artemisinin-based combinations, the establishment of rapid diagnostic tests (RDTs), the implementation of a village malaria health worker system, and the widespread use of long-lasting insecticidal bed nets (LLINs) [2–5].

Though its incidence declines significantly, malaria continuously poses a major burden on public health in this nation, in which there is a co-occurrence of *Plasmodium vivax* and *Plasmodium falciparum*. However, *P. vivax* control is obviously less effective [6]. Cases of *P. vivax* have major distributions crossing six northeastern Cambodia provinces (20,954, ~ 80%), and particularly prevail in Stung Treng within the Cambodia-Laos border (~ 28% of overall vivax case identified) [7].

The transmission of malaria in the GMS is characterized by vector diversity and a high degree of spatial heterogeneity in the distribution pattern [8]. Generally, *Anopheles minimus*, *Anopheles dirus* complex, and *Anopheles sinensis* refer to the major vector, whereas the significance pertaining to the respective species in the malaria-transmitting process changes extensively with areas [9]. Some thorough investigations were conducted in the GMS, unfortunately, most of these researches failed to screen for “secondary” vectors (e.g., *Anopheles nivipes*) in terms of *Plasmodium* infection [10–12]. *Anopheles nivipes* was found to be *Plasmodium* positive according to a study in Bangladesh in 2012 [13] and has also been reported positive for *Plasmodium* parasites in India and other countries [14, 15]. In Cambodia, *An. nivipes* was a secondary vector, as well as *Anopheles philippinensis* [9] in transmitting malaria and had been long suspected.

On the other hand, though mosquito population exhibiting various gene makeups is likely to be different in vector competence, rare data has been achieved regarding population genetics of *An. nivipes* in the Cambodia-Laos border. Data regarding genetic diversity and population structure can help develop available mosquito control plans [16, 17]. Owing to its significant sensitivity in the field of system and phylogenetic relationships for maternal inheritance and haploidization [18], Mitochondrial DNA (mtDNA) has been widely used as an effective genetic marker in describing molecular taxonomy, phylogenetic relationships, population structure, and genetic diversity in malaria vectors [19].

Given the existing drastic variations of malaria conditions in Cambodia, this work aimed to study the mosquito diversity and molecular phylogeny of secondary vectors i.e., *An. nivipes* in the Cambodia-Laos border. Siem Pang County (Stung treng Province) was taken as the investigation site and mosquito surveillance was firstly performed. The cytochrome c oxidase subunit 2 gene (COII) of mtDNA from *An. nivipes* samples were subsequently sequenced. Furthermore, the gene variability and phylogenetic relationship of *An. nivipes* along the Cambodia-Laos border were investigated and compared with other samples from India, Thailand, and Myanmar for examining their gene relations. This work largely aimed at gaining more insights into the continuous surveillance of mosquito vectors, as well as molecular phylogeny and evolution of *An. nivipes* in the Cambodia-Laos border by using an effective genetic marker, mtDNA-COII.

## Methods

### Study site

Stung treng borders Laos in the north and is located on the east bank of the Mekong River. This endemic region takes up nearly 11,000 square kilometres and has about 42,000 residents. The average population density is 3.82 people per square kilometre. It has dense forests, diversified landforms, the temperature changes greatly and the annual rainfall is 1800 mm.

### Mosquito collection and identification

Adult mosquitoes were collected in two villages in Siem Pang County by overnight trapping with the battery-operated CDC light traps (Model: 1012, Origin: John W. Hock Inc, USA) in the cattle/pig pens or human rooms from 8:00 pm to 08:00 am and continued for 1–5 nights, in 2018. Four CDC light traps were operating in two cattle/pig pens and two human rooms each night. All live adult mosquitoes were killed by freezing in the refrigerator and subsequently isolated and distinguished according to subgroup, species, and sex through dissecting microscope based on standards procedures in the field office of Siem Pang County [20, 21]. All mosquitoes were initially morphologically sorted out in the field using the keys of Das et al. [22]. Each morphologically identified specimen was kept individually in capped plastic beam capsules having silica gel and stored at 4 °C for molecular species confirmation and further processing. To avoid any deviations in the further analysis, molecular identification of *An. nivipes* based on COII was subsequently performed. Final species confirmation is required to have ≥ 98% sequence identity to the voucher specimens/sequences in the NCBI nt databases. To avoid the issue that COII alone did not produce significant results for the voucher sequence, the amplification and sequencing on ITS2 was also performed. As a result, ITS2 and COII database comparisons of each morphologically identified *An. nivipes* samples were paired to determine the species of *An. nivipes* (Additional file 1: Table S1).

### DNA extraction and sequencing

The extraction of *An. nivipes* genomic DNA in individual mosquitoes was carried out following the producer’s manual (QIAamp^®^ DNA Mini Kit, Germany). The amplification for approximately 620 bp of COII gene was carried out using primers, LYS-R (5′-ACTTGCTTTCAGTCATCTAATG-3′) and LEU-F (5′-TCTAATATGGCAGATTAGTGCA-3′). The overall PCR reaction volume reached 20 µl and the mixture of PCR reagent comprised 2 µl of DNA, 0.05 unit of Takara Taq (Dalian, China), 0.3 µM of the respective primer, 0.2 mM of dNTPs, and 2.5 µl of 10× Buffer (15 mM MgCl2, 100 mM Tris–HCl ^PH=8.3^ as well as 500 mM KCl). Besides, the cycling parameter included 95 °C, 5 min; 95 °C/1 min, 51 °C/1 min, 72 °C/2 min for 35 cycles; with a final extension of 72 °C for 10 min. The PCR products were analysed by 1.5% agarose gel electrophoresis stained with GoldView (Solarbio, China), under UV transillumination. The sequencing reaction proceeded in both directions using an ABI Big Dye Terminator Kit (Applied Biosystems, Thermo Fisher Scientific) and was analysed through ABI Prism 3500xL Genetic Analysis Tool (Applied Biosystems, Thermo Fisher Scientific) in Shanghai (Sangon Biotech).

### Sequence alignment and phylogenetic analysis

In order to analyse the sequence variations and genetic relationship of *An. nivipes* from Cambodia-Laos and neighbouring nations, multi-sequence alignment of COII sequence was firstly carried out after the retrieval of sequence deposited in GenBank by using the keywords “(species name) & COII” (http://www.ncbi.nlm.nih.gov/). The manual adjustment was conducted by using BioEdit V7.0.9 if required [23]. Gaps were excluded from the analysis and characters were unweighted. Subsequently, a phylogenetic tree was generated using a Neighbour-joining algorithm bootstrapped with 1000 replicates [24] based on MEGA-X [25]. The visualization of this phylogram was performed with the use of Figtree v1.4.2 [26]. *Anopheles lindesayi* (GenBank Accession No. AJ620898) and *Anopheles claviger* (GenBank accession nos. AY129232 and DQ229313) were used as outgroup taxa to the *An. nivipes*, following previous studies [27].

### Statistical analysis

The average of nucleotide differences per site (K), nucleotide diversity (π), haplotype diversity (Hd), and haplotypes (H) was calculated by using DnaSP v.5.0 [28]. For more widely comparing haplotypes in Cambodia-Laos and other geographical regions, the existing data in GenBank from China, India, and Thailand were analysed, and a parsimony framework was subsequently carried out by using Network 4.0 [29].

The calculation of pairwise *F*_ST_ for estimating population differentiation by complying with a difference in haplotype frequency, Nei’s Nm estimated gene flow conformed to *G*_ST_ [30], as well as analysis of molecular variance (AMOVA) for determining the distribution of genetic variation in population using 1000 permutations were performed using Arlequin v.3.5 [31].

Moreover, a spatial analysis of molecular variance (SAMOVA 2.0) was conducted for clustering COII sequences to homogeneous populations with genetic and geographical homogeneousness [32]. SAMOVA generates F-statistics (*F*_CT_, *F*_ST_, *F*_SC_), with the use of the AMOVA approach, to K group for maximizing group variations. SAMOVA estimate was calculated for K = 2–8, with 1000 simulated annealing steps from each of 100 sets of initial starting conditions. Furthermore, isolation by distance (IBD) was examined using a nonparametric Mantel with the web-based computer program IBDWS v.3.16 [33].

Using the statistics D [34] and Fu’s Fs [35], the hypothesis of strict neutrality was tested in *An. nivipes* population based on DnaSP v.5.0. The mismatch distribution (simulated in Arlequin v.3.5) was carried out for distinguishing smooth unimodal distribution from multimodal or ragged distribution [36–38]. For rejecting the demographic expansion hypothesis, the difference with statistical significance in distribution under the observation and simulation was assessed with the sum of square deviations (SSD).

## Results

### Surveillance of mosquito abundance and composition

**Fig. 1:**
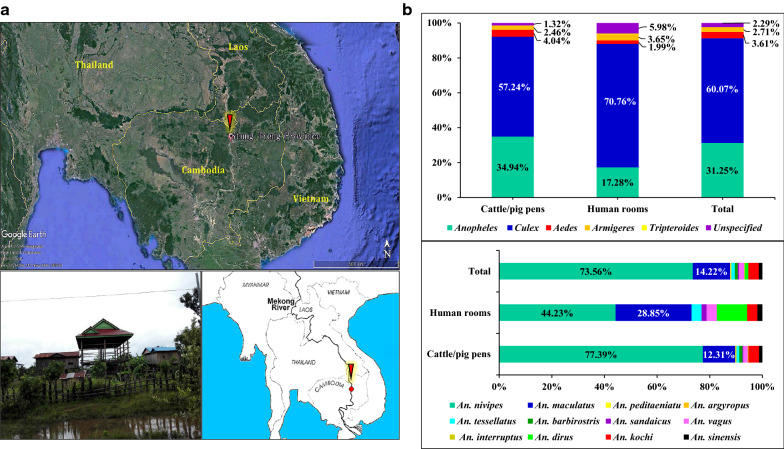
Sampling sites and diversity of adult mosquitoes in Cambodia-Laos border. **a** The red label indicated the sampling site in Siem Pang County (Stung treng Province, 14°17′ N, 106°23′ E). The map was prepared by using Google Earth Pro (7.3.0.3832). **b** The pie charts showed the genus composition of adult mosquitoes and species composition of *Anopheles* mosquitoes collected from Human rooms and/or Cattle/pig pens. All the adult mosquitoes were trapped using overnight trapping with the battery-operated CDC light traps (Model: 1012, Origin: John W. Hock Inc, USA)

A total of 1440 adult mosquitoes were collected in cattle/pig pen or human rooms through overnight trapping with the battery-operated CDC light traps (Fig. 1a). Those mosquitoes were classified into 5 genera including *Anopheles*, *Culex*, *Aedes*, *Armigeres*, *Tripteroides,* and 27 species. The predominant genera included *Culex* (9 species) and *Anopheles* (12 species), respectively taking up 60.07% (865/1440) and 31.25% (450/1440); Other genera including *Aedes*, *Armigeres*, and *Tripteroides* respectively accounted for 3.61% (52/1440), 2.71% (39/1440), and 0.07% (1/1440); In addition, undefined genera/species accounted for 2.29% (33/1400) (Fig. 1b, Additional file 2: Table S2). Among the Genus *Anopheles*, *An. nivipes,* and *An. maculatus* were two major species and accounted for 73.56% (331/450) and 14.22% (64/450), respectively, while *Anopheles peditaeniatus*, *Anopheles argyropus*, *Anopheles tessellatus*, *Anopheles barbirostris*, *Anopheles sandaicus*, *Anopheles vagus*, *Anopheles interruptus*, *An. dirus*, *Anopheles kochi*, and *An. sinensis* accounted for merely 0.22% (1/450), 0.22% (1/450), 1.33% (6/450), 1.11% (5/450), 0.44% (2/450), 2.00% (9/450), 0.22% (1/450), 1.33% (6/450), 4.00% (18/450), and 1.33% (6/450) (Fig. 1b, Additional file 2: Table S2).

### Genetic diversity of *Anopheles nivipes* among populations

DNA samples were extracted from 53 mosquitos which were collected in Siem Pang and morphologically/molecularly identified as *An. nivipes* (Additional file 1: Table S1). The amplification of the COII sequence was achieved in these 53 specimens, representing populations from the Cambodia-Laos border. To draw a broader comparison in haplotype from Cambodia-Laos and other geographical regions, the available data in GenBank from neighbouring nations were downloaded and analysed (Additional file 3: Table S3). In general, a total of 86 *An. nivipes* COII sequences were generated for 10 populations, including Siem Pang (n = 53), Tripura (India; n = 4), Cheema (Punjab, India; n = 2), Bathinda (Punjab, India; n = 2), Nagaland (India; n = 6), Assam (India; n = 7), Myanmar (n = 2), Thailand (n = 2), Mizoram (India; n = 2) and Meghalaya (India; n = 6) (Table 1, Additional file 4: Fig. S1). A high level of nucleotide diversity (Pi = 0.0156), number of haplotypes (h = 47), and haplotype diversity (Hd = 0.9237) were observed in COII populations (Table 1).

**Table 1:** Genetic diversity indices and neutrality tests (Fu’s *Fs* and Tajima’s *D*) based on the COII gene of *An. nivipes*

Species	n	Haplotype code	S	Pi	h	Hd	k	Fu's *Fs*	Tajima's *D*
Total	86	H1(19), H2(13), H3(1), H4(1), H5(1), H6(8), H7(1), H8(1), H9(1), H10(1), H11(1), H12(2), H13(1), H14(1), H15(1), H16(1), H17(1), H18(1), H19(1), H20(1), H21(1), H22(1), H23(1), H24(1), H25(1), H26(1), H27(1), H28(2), H29(1), H30(1), H31(1), H32(1), H33(1), H34(1), H35(1), H36(1), H37(1), H38(1), H39(1), H40(1), H41(1), H42(1), H43(1), H44(1), H45(1), H46(1), H47(1)	223	0.01560	47	0.92370	8.12558	− 19.201***	− 2.39879***
KH_St_Sp	53	H1(19), H2(12), H3(1), H4(1), H5(1), H6(8), H7(1), H8(1), H9(1), H10(1), H11(1), H12(2), H13(1), H14(1), H15(1), H16(1)	19	0.00243	16	0.80700	1.50363	− 10.944***	− 2.01237**
IN_Tri	4	H24(1), H25(1), H26(1), H27(1)	8	0.00701	4	1.00000	4.33333	− 0.715	− 0.06867
IN_Pun_Ch	2	H21(1), H22(1)	13	0.01268	2	1.00000	7.00000	1.946	n.d
IN_Pun_Ba	2	H20(1), H23(1)	65	0.04415	2	1.00000	23.00000	3.135	n.d
IN_Nag	6	H36(1), H37(1), H38(1), H39(1), H40(1), H41(1)	14	0.01079	6	1.00000	6.66667	− 1.623	0.53608
IN_Ass	7	H33(1), H34(1), H35(1), H44(1), H45(1), H46(1), H47(1)	28	0.02350	7	1.00000	14.52381	− 0.938	0.09554
MM	2	H17(1), H18(1)	2	0.00324	2	1.00000	2.00000	0.693	n.d
TH	2	H2(1), H19(1)	3	0.00485	2	1.00000	3.00000	1.099***	n.d
IN_Miz	2	H42(1), H43(1)	3	0.00485	2	1.00000	3.00000	1.099***	n.d
IN_Meg	6	H28(2), H29(1), H30(1), H31(1), H32(1)	11	0.00744	5	0.93330	4.60000	− 0.496	− 0.27307

n.d., not determined; n.s., *P* > 0.10; ^#^*P* < 0.10; *P < 0.05; **P < 0.02; ***P < 0.001. KH_St_Sp, Siem Pang County (Stung treng, Cambodia); IN_Tri, Tripura (India); IN_Pun_Ch, Cheema (Punjab, India); IN_Pun_Ba, Bathinda (Punjab, India); IN_Nag, Nagaland (India); IN_Ass, Assam (India); MM, Myanmar; TH, Thailand; IN_Miz, Mizoram (India); IN-Meg, Meghalaya (India)

*n* number of sequences, *S* number of polymorphic sites, *pi* nucleotide diversity, *h* number of haplotypes, *Hd* haplotype diversity

### Population structure and genetic differentiation

**Fig. 2:**
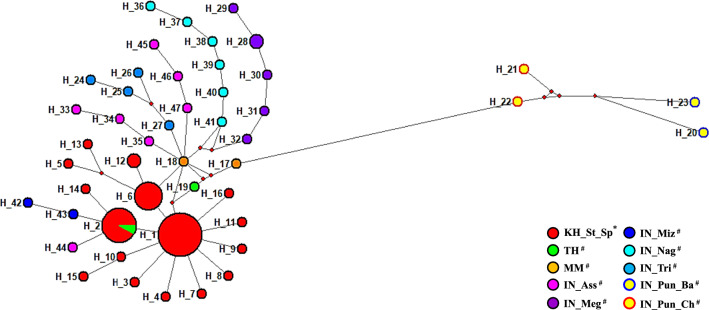
Phylogenetic network of 47 mitochondrial haplotypes of the COII gene in *Anopheles nivipes*. Localities are indicated by different colours (bottom-right). The area of each circle is approximately proportional to the frequency of the haplotype. ^#^Samples available in Genbank. *Samples from Cambodia-Laos border. KH_St_Sp, Siem Pang County (Stung treng, Cambodia); IN_Tri, Tripura (India); IN_Pun_Ch, Cheema (Punjab, India); IN_Pun_Ba, Bathinda (Punjab, India); IN_Nag, Nagaland (India); IN_Ass, Assam (India); MM, Myanmar; TH, Thailand; IN_Miz, Mizoram (India); IN_Meg, Meghalaya (India)

The median-joining network based on 86 COII sequences denoted the distribution pattern exhibited by 47 haplotypes in *An. nivipes* population. The *An. nivipes* populations fell into three Groups. Group 1 consisted of all the haplotypes except for Bathinda and Cheema; Group 2 consisted of haplotype from Cheema (Punjab, Northwest India); Group 3 with haplotype from Bathinda (Punjab, Northwest India). The most common haplotypes referred to H1 (n = 19), H2 (n = 13), and H6 (n = 8), as only identified in 73.58% (39/53) of Siem Pang and 50% (1/2) of Thailand. H21 (n = 1), H22 (n = 1) was only identified in Cheema; H20 (n = 1) and H23 (n = 1) were only identified in Bathinda (Fig. 2, Additional file 5: Fig. S2). In addition, a single individual denoted other 38 haplotypes but derived from haplotype 1 through a very few mutation steps. The UPGMA dendrogram based on K2P genetic distances between haplotypes indicated that H21, H22 constituted one cluster, H20 and H23 constituted another, while the other haplotypes constituted the third (Additional file 6: Fig. S3).

**Fig. 3:**
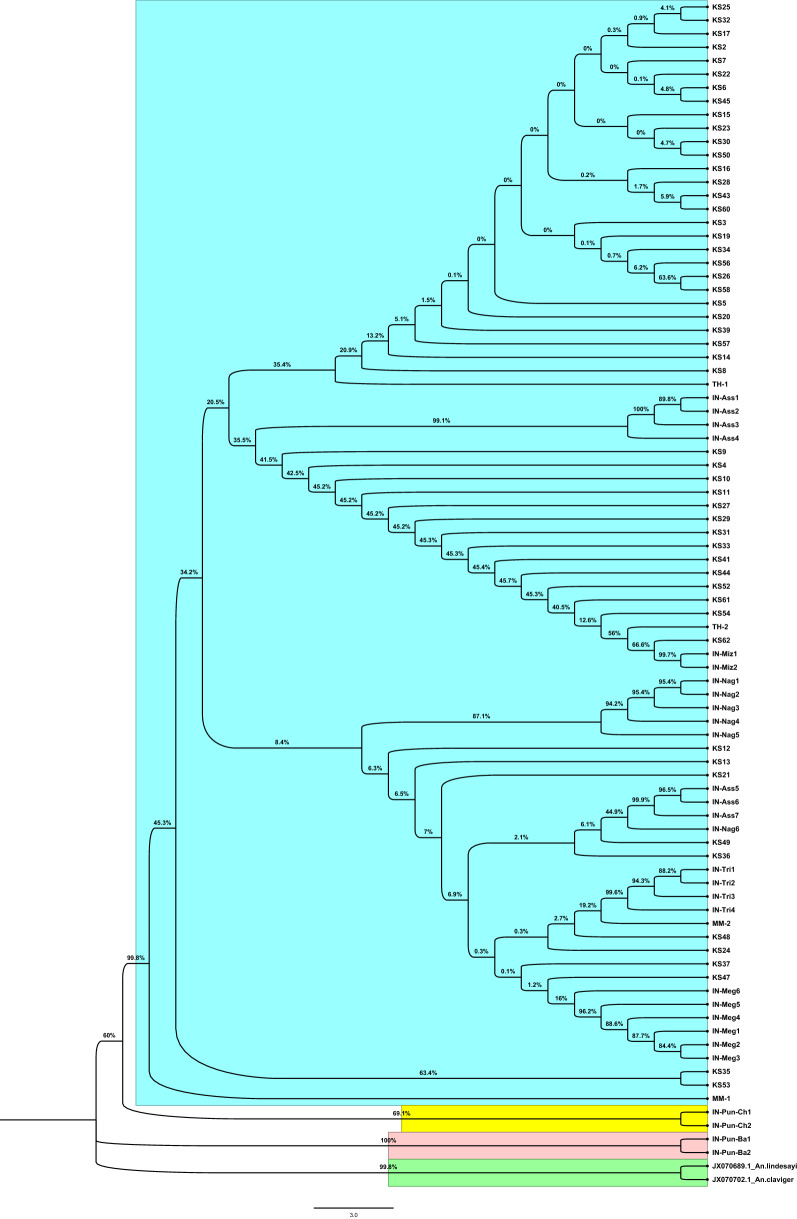
Neighbour-joining phylogenetic tree of *Anopheles nivipes* based on COII sequences from GenBank and our original data. Bootstrap values (1000 replicates) of neighbour-joining analyses are shown above/below the main lineages. Lineage designation is indicated on the right. Bars represent 3.0 substitutions per site based on COII. Different colours indicated different population groups of *An. nivipes*. *An. lindesayi* and *An. claviger* were used as the outgroup taxa. KS, Siem Pang County (Stung treng, Cambodia); IN_Tri, Tripura (India); IN_Pun_Ch, Cheema (Punjab, India); IN_Pun_Ba, Bathinda (Punjab, India); IN_Nag, Nagaland (India); IN_Ass, Assam (India); MM, Myanmar; TH, Thailand; IN_Miz, Mizoram (India); IN_Meg, Meghalaya (India)

AMOVA analysis based on COII sequences demonstrated that most of the variances were found among group variation (86.72%) rather than within populations (4.56%) and among populations within groups (8.72%), suggesting that these populations could fall to several groups. The fixation index among groups (*F*_CT_), among populations within groups (*F*_SC_), and within populations (*F*_ST_) showed statistical significance (*P* < 0.05) (Table 2). The neighbour-joining dendrogram indicated that the populations of Siem Pang (Stung treng, Cambodia), Tripura (Northeast India), Nagaland (Northeast India), Assam (Northeast India), Myanmar, Thailand, Mizoram (Northeast India), and Meghalaya (Northeast India) were clustered in one clade, whereas Cheema (Punjab, Northwest India) and Bathinda (Punjab, Northwest India) formed the second and third clades (Fig. 3). *Anopheles lindesayi* and *An. claviger* were introduced as outgroups which clustered into another clade.

**Table 2:** Analysis of molecular variance (AMOVA) of ten *An. nivipes* populations based on COII

Source of variation	d. f	Sum of squares	Variance components	% of variation	Fixation index (*P*)
Among groups	2	336.833	40.15792 Va	86.72	*F*_CT_: 0.86722 (*P* < 0.05)
Among populations within groups	7	200.265	4.03898 Vb	8.720	*F*_SC_: 0.65689 (*P* < 0.05)
Within populations	76	160.332	2.10964 Vc	4.560	*F*_ST_: 0.95444 (*P* < 0.05)
Total	85	697.430	46.30654		

*F*_CT_ Fixation index among groups; *F*_SC_ among populations within groups; *F*_ST_ within populations

The maximal level of genetic differentiation in accordance with the fixation index *F*_ST_ based on sequences analysis of COII was between Cheema and Siem Pang (*F*_ST_ = 0.97824, *P* < 0.05). Estimates of gene flow (Nm) varied extensively in populations, ranging from 0.01112 to 8.44874. The minimal was between Cheema and Siem Pang (Nm = 0.01112). The maximal was between Assam and Myanmar (Nm = 8.44874) (Additional file 7: Table S4).

### Spatial genetic structure analysis

**Fig. 4:**
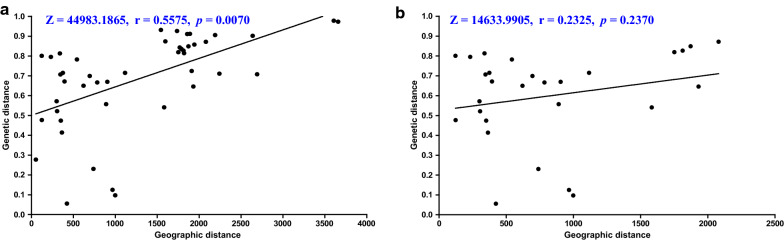
Isolation by distance, the relationship between geographical and genetic distances based on COII sequences in *Anopheles nivipes* populations. Isolation by distance (IBD) was examined using a nonparametric Mantel with the web-based computer program IBDWS v.3.16. **a** All populations based on COII sequences; **b** All populations without Punjab samples based on COII sequences

According to the COII-based SAMOVA analysis, the clustering number was between 2 and 9. High genetic differentiation between groups was detected, as the *F*_CT_ value ranged from 0.74758 to 0.867 and was statistically significant. *F*_CT_ value was maximal at K = 3 (0.867; *P* < 0.05) with *F*sc (0.65541, *P* < 0.001). In this case, two Punjab populations (Bathinda and Cheema) became separated and assigned to two single population groups, leaving other populations, which demonstrated that there is a significant genetic barrier to restrict gene flow from Punjab to other populations. As K value increased up to K = 8, Siem Pang and Thailand remained attributed to one group, whereas the other population created 8 distinct groups (Table 3). In general, the patterns identified in SAMOVA analyses complied with the phylogenetic trees (Fig. 3). The UPGMA dendrogram based on Nei’s unbiased genetic distance between populations revealed three distinctive groups, as Bathinda and Cheema constituting two groups, and the remaining populations covered in a third group (Additional file 8: Fig. S4). A Mantel test revealed a significant correlation between geographical and genetic distances in all populations (Z = 44,983.1865, r = 0.5575, *P* = 0.0070), suggesting that the genetic structure observed in *An. nivipes* population could be partially explained by distance isolation based on COII sequence analysis (Fig. 4).

**Table 3:** Population groups identified by spatial analysis of molecular variance (SAMOVA) algorithm based on COII

K	Population grouping	*F*_CT_	*F*_SC_
k = 2	[IN_Pun_Ba, IN_Pun_Ch][KH_St_Sp, MM, TH, IN_Tri, IN_Meg, IN_Ass, IN_Nag, IN_Miz]	0.84809*	0.68162***
k = 3	[IN_Pun_Ba][IN_Pun_Ch][KH_St_Sp, MM, TH, IN_Tri, IN_Meg, IN_Ass, IN_Nag, IN_Miz]	0.867*	0.65541***
k = 4	[IN_Pun_Ba][IN_Pun_Ch][KH_St_Sp, MM, TH, IN_Tri, IN_Meg, IN_Ass, IN_Nag][IN_Miz]	0.81663**	0.65331***
k = 5	[IN_Pun_Ba][IN_Pun_Ch][KH_St_Sp, TH, IN_Tri, IN_Meg, IN_Ass, IN_Nag][MM][IN_Miz]	0.75603**	0.66771***
k = 6	[IN_Pun_Ba][IN_Pun_Ch][IN_Tri][IN_Meg][IN_Nag][KH_St_Sp, TH, IN_Ass, MM, IN_Miz]	0.74758*	0.48268***
k = 7	[IN_Pun_Ba][IN_Pun_Ch][IN_Tri][IN_Meg][IN_Nag][IN_Ass][KH_St_Sp, TH, MM, IN_Miz]	0.7595*	0.35732***
k = 8	[IN_Pun_Ba][IN_Pun_Ch][IN_Tri][IN_Meg][IN_Nag][IN_Ass][KH_St_Sp, TH, MM][IN_Miz]	0.82893**	0.06355**
k = 9	[IN_Pun_Ba][IN_Pun_Ch][IN_Tri][IN_Meg][IN_Nag][IN_Ass][KS,TH][MM][IN_Miz]	0.85191*	− 0.12893^ns^

Significant values ^*^P < 0.05; ^**^P < 0.01; ^***^P < 0.001, ns: Not significant

KH_St_Sp, Siem Pang County (Stung treng, Cambodia); IN_Tri, Tripura (India); IN_Pun_Ch, Cheema (Punjab, India); IN_Pun_Ba, Bathinda (Punjab, India); IN_Nag, Nagaland (India); IN_Ass, Assam (India); MM, Myanmar; TH, Thailand; IN_Miz, Mizoram (India); IN_Meg, Meghalaya (India)

### Demographic history and neutrality test

As indicated from Tajima’s D and Fu’s Fs tests based on COII, the Siem Pang population exhibited significant negativity (*P* < 0.05, *P* < 0.02), suggesting a recent population expansion or selection (Table 1). The observed smooth and unimodal mismatch distribution suggested a sudden population expansion, conforming to the mismatch distribution derived under the model of sudden expansion (Additional file 9: Fig. S5).

## Discussion

The spread of malaria in GMS exhibits the diversity of vector species and the high spatial heterogeneity of distribution patterns [8]. In 2002 about 61% of Cambodia’s total land area was estimated to be forested [39] with over 80% of that in malaria-endemic areas [40]. These forested areas are primarily located in provinces bordering Vietnam, Laos, and Thailand. Efficient forest malaria vectors are found, including *An. dirus* and *An. minimus*, so a high risk of malaria is imposed on people living in villages on the edge of the forest or engaged in forest activities [41–44]. Besides the main forest vector, considerable other *Anopheles* species appear close to human settlements. Under high coverage of insecticide-treated nets, transmission by “secondary” vectors with outdoor or early biting behaviour may be more important than transmission by primary vectors [45], since the secondary vectors are less anthropogenic and may be more prone to exophagous and early bites. Furthermore, the exogenous incubation period of *P. vivax* is highlighted to be shorter, and the secondary vector is likely to be a more effective vector as compared with *P. falciparum* [46]. There is a need to study the extent to which malaria vectors in Cambodia are outdoor bites and early bites, and to assess the importance of secondary vectors.

Several studies have been conducted to screen the secondary or suspected vector in the GMS. In central Laos (Khammouane), *An. nivipes* was one of the predominant species and accounted for 11.55% [47], while in the south-eastern part of Laos (Nongceng), instead of the major vector *An. minimus* and *An. dirus*, *An. nivipes* is suspected to be the dominant vector, taking up over 65% [48]. In Thailand, *An. nivipes*, as well as *An. philippinensis*, were suspected vectors and accounted for 30.18% and 27.32%, respectively [49]. *Anopheles nivipes* referred to a secondary vector, as well as the *An. philippinensis* in Cambodia [9]. According to St Laurent B’s research [15], *An. nivipes* referred to one of the five most prevalent species and accounted for 23.49% in Preah Vihear and 35.61% in Ratanakiri Provinces, both located in the north Cambodia and border with Stung treng Province. Likewise, in this work, *An. nivipes* accounted for 22.99% (331/1440) in Siem Pang County of Stung treng (Fig. 1, Additional file 2: Table S2). It is noteworthy that *An. nivipes* has been reported positive for *Plasmodium* parasites in Bangladesh, India, and other countries [13–15]. Besides, the vivax malaria case was largely reported in three northern provinces, Preah Vihear, Ratanakiri, and Stung treng, suggesting that *An. nivipes* might be a potential and important vector of *P. vivax* in northern Cambodia [7].

In mosquito barcoding studies, the most frequently used molecular markers or barcoding region is the cytochrome c oxidase subunit I (COI) of the mitochondrial genome (mtDNA), followed by the internal transcriptional spacer 2 (ITS2) of ribosomal DNA (rDNA). Since mtDNA is typically inherited maternally, any hybrid or offspring would only have the mtDNA from the maternal species [50]. Furthermore, mitochondrial genomes evolve 5–10 times faster than nuclear genomes, making mtDNA potentially more useful than rDNA in correctly identifying recently differentiated species [51, 52]. Some researchers used COI or COII as the only marker to identify mosquito species and study their molecular evolution [16, 53–58]. Additionally, due to the typically high copy numbers, availability of conserved primer binding sequences, and ease of amplification [19], variation in mitochondrial DNA (mtDNA) is being used more recently to approach levels of population structure and genetic diversity within species [59]. Nonetheless, more reliable species information can be obtained if multiple molecular markers are used simultaneously [60–62]. Based on mtDNA-COII sequencing in this work, the AMOVA analysis indicated that all populations could be divided into different groups (Table 2). According to phylogenetic analysis, the *An. nivipes* could fall to two Northwest India groups (Bathinda, Cheema) and a Northeastern India/SEA group (Figs. 2, 3). As revealed from strong genetic differentiation and limited gene flow between the Cambodia-Laos and Northwest India populations, these regions might undergo genetic isolation (Additional file 7: Tables S4). SAMOVA analysis further suggested a genetic barrier restricting gene flow between these populations (Table 3), and the patterns were also consistent with the phylogenetic trees. The Bay of Bengal, in the northeast part of the Indian Ocean, is situated between India to the west and northwest, Bangladesh to the north, and Myanmar to the east, acting as a natural barrier to interrupt the spread of malaria vector. On the contrary, the weak genetic differentiation and frequent gene flow between the Cambodia-Laos and other populations such as Northeast India, Myanmar, and Thailand suggest that these regions are not genetically isolated. Moreover, the Mantel test revealed a strong correlation of genetic and geographical distances, which demonstrated the significant isolation by the distance of *An. nivipes* (Fig. 4). Unimodal plots of the mismatch distribution were observed in the Cambodia-Laos population (Additional file 9: Fig. S5), as well as the Tajima’s *D* and Fu’s *Fs* test were both negative and significant, thereby demonstrating a recent expansion after a bottleneck of *An. nivipes* population in this border region (Table 1).

In the present study, the molecular phylogeny analysis of *An. nivipes* based on ITS2 was also conducted to evaluate its effectiveness compared to mtDNA-COII (Additional file 10). Specific to ITS2, no genetic barriers were identified between the Cambodia-Laos and other populations. SAMOVA analysis and Mantel test revealed no correlation between genetic and geographical distances, thereby demonstrating no isolation-by-distance was found in *An. nivipes* populations based on ITS2 (Additional file 10). Since ITS2 regions alone have been used in distinguishing closely related mosquito species belonging to various genera such as *Anopheles* [17], Culex [63], and Aedes [64], mtDNA-COII may be a more effective marker than rDNA-ITS2 for describing the genetic diversity and population structure of *An. nivipes* due to its advantages of maternal inheritance, no recombination, and high variability [62].

## Conclusion

This work reported that a high diversity of mosquito vectors was found and *Anopheles nivipes* was one of the major *Anopheles* species in the Cambodia-Laos border. A genetic barrier limiting gene flow between Cambodia-Laos and two Northwest India populations had been found based on sequences analysis of mtDNA-COII, and a recent population expanding or selecting of *An. nivipes* occurred in this border, suggesting that mtDNA-COII can serve as effective markers for describing the genetic diversity and population structure of *An. nivipes.* Further investigation and continuous surveillance of *An. nivipes* are warranted in this region.

## Supplementary Information

**Additional file 1: Table S1.** Full list of 53 *Anopheles nivipes* specimens which were classified by both molecular and morphological identifications, with morphology species ID, molecular species ID based on ITS2, molecular species ID based on COII, geographical location, latitude, and longitude.

**Additional file 2: Table S2.** Genus/Species compositions of mosquitoes trapped by CDC lamp in SIEM PANG County, Stung treng Province. Adult mosquitoes were collected by using overnight trapping with the battery-operated CDC light traps. ^**^ indicated the genus or species composition of mosquitoes which were described in percentage. (%)^**^ indicated the genus or species composition of mosquitoes which were described in numbers.

**Additional file 3: Table S3.** COII sequences of *Anopheles nivipes* were downloaded from the NCBI. ^*^ indicated the longitude and latitude coordinates to the geographical center of a certain province, due to the samples being initially collected from various sampling sites in a certain province. *An. niv*, *Anopheles nivipes*.

**Additional file 4: Figure S1.** Map of the populations from different geographical regions. Populations of mitochondrial COII sequences: KH, Siem Pang County (Stung treng, Cambodia); Tri, Tripura (India); Ch, Cheema (Punjab, India); Ba, Bathinda (Punjab, India); Nag, Nagaland (India); Ass, Assam (India); MM, Myanmar; TH, Thailand; Miz, Mizoram (India); Meg, Meghalaya (India). The map was prepared by using LocaSpace Viewer.

**Additional file 5: Figure S2.** Distribution heatmap of haplotype based on COII. KH-St-Sp, Siem Pang County (Stung treng, Cambodia); MM, Myanmar; TH, Thailand; IN-Pun-Ba, Bathinda (Punjab, India); IN-Pun-Ch, Cheema (Punjab, India); IN-Tri, Tripura (India); IN-Meg, Meghalaya (India); IN-Ass, Assam (India); IN-Nag, Nagaland (India); IN-Miz, Mizoram (India). The numbers of haplotypes are shown on the right side of the figure. The color scale ranges from blue to red, showing a range from minimum number (0) to maximum numbers (19) for each haplotype.

**Additional file 6: Figure S3.** Neighbor-joining phylogenetic tree of *An. nivipes* haplotypes based on COII sequences from GenBank and original data in this study. Bootstrap values (1000 replicates) of Neighbor-Joining analyses are shown above/below the main lineages. Lineage designation is indicated on the right. Bars represent 2.0 substitutions per site based on COII. Different colors indicated different population groups of *An. nivipes*.

**Additional file 7: Table S4.** Genetic differentiation and Gene flow among the Geographic Groups based on COII. The pairwise *F*_ST_ values and Nm values based on the COII are shown below and above the diagonal, respectively. Characters in bold indicate the significance (*P* < 0.05). inf, infinite. KH_St_Sp, Siem Pang County (Stung treng, Cambodia); IN_Tri, Tripura (India); IN_Pun_Ch, Cheema (Punjab, India); IN_Pun_Ba, Bathinda (Punjab, India); IN_Nag, Nagaland (India); IN_Ass, Assam (India); MM, Myanmar; TH, Thailand; IN_Miz, Mizoram (India); IN-Meg, Meghalaya (India).

**Additional file 8: Figure S4.** Cluster analysis based on COII sequences in *Anopheles nivipes* populations. UPGMA dendrogram based on Nei ‘s unbiased genetic distance between different populations of *An. nivipes*. Bars represent 0.7 substitutions per site based on COII. KS, Siem Pang County (Stung treng, Cambodia); IN_Tri, Tripura (India); IN_Pun_Ch, Cheema (Punjab, India); IN_Pun_Ba, Bathinda (Punjab, India); IN_Nag, Nagaland (India); IN_Ass, Assam (India); MM, Myanmar; TH, Thailand; IN_Miz, Mizoram (India); IN_Meg, Meghalaya (India).

**Additional file 9: Figure S5.** Mismatch distribution graphs for Siem Pang population. The X and Y-axis show the number of pairwise differences and the frequency of the pairwise comparisons, respectively. The observed frequencies are represented by a dotted line. The frequency expected under the hypothesis of the constant population model is depicted by a solid line. **(a)** all populations-COII; **(b)** Siem Pang population-COII.

**Additional file 10. **Supplementary data of molecular phylogeny data based on ITS2. This file contains Tables 1–5 and Figs. 1–9.

## Data Availability

Data supporting the conclusions of this article are included within the article and its additional files. The datasets generated and/or analysed during the current study are available in the GenBank (http://www.ncbi.nlm.nih.gov/). The raw datasets used and/or analysed during this study are available from the corresponding author upon reasonable request.

## Abbreviations

GMS: Greater Mekong subregion
RDT: Rapid diagnostic tests
LLITNs: Long-lasting insecticide-treated bed nets
ITS2: Internal transcribed spacer 2
COII: Cytochrome c oxidase subunit 2
AMOVA: Analysis of molecular variance
SAMOVA: Spatial analysis of molecular variance
IBD: Isolation by distance
mtDNA: Mitochondrial genome
