# Supplementary material for: Molecular phylogeny of Anopheles nivipes based on mtDNA-COII and mosquito diversity in Cambodia-Laos border
Source: Malar J. 2022 Mar 17;21:91. doi: 10.1186/s12936-022-04121-w (PMC8932176; doi:10.1186/s12936-022-04121-w)
Supplement: Supplementary file 1 — Additional file 1: Table S1. Full list of 53 Anopheles nivipes specimens which were classified by both molecular and morphological identifications, with morphology species ID, molecular species ID based on ITS2, molecular species ID based on COII, geographical location, latitude, and longitude. [file 12936_2022_4121_MOESM1_ESM.docx]

**Table S1. Full list of 53 *Anopheles nivipes* specimens collected in SIEM PANG County, Stung treng Province.**

| **No.** | **Morphology species ID** | **Molecular species ID based on ITS2** | **Molecular species ID based on COII** | **Location** | **Latitude** | **Longitude** |
| --- | --- | --- | --- | --- | --- | --- |
| 1 | *An. nivipes KS1* | *An. nivipes KS1* | */* | Siem Pang, Stung treng, Cambodia | 106°23'N | 14°17'E |
| 2 | *An. nivipes KS2* | *An. nivipes KS2* | *An. nivipes KS2* | Siem Pang, Stung treng, Cambodia | 106°23'N | 14°17'E |
| 3 | *An. nivipes KS3* | *An. nivipes KS3* | *An. nivipes KS3* | Siem Pang, Stung treng, Cambodia | 106°23'N | 14°17'E |
| 4 | *An. nivipes KS4* | *An. nivipes KS4* | *An. nivipes KS4* | Siem Pang, Stung treng, Cambodia | 106°23'N | 14°17'E |
| 5 | *An. nivipes KS5* | *An. nivipes KS5* | *An. nivipes KS5* | Siem Pang, Stung treng, Cambodia | 106°23'N | 14°17'E |
| 6 | *An. nivipes KS6* | *An. nivipes KS6* | *An. nivipes KS6* | Siem Pang, Stung treng, Cambodia | 106°23'N | 14°17'E |
| 7 | *An. nivipes KS7* | *An. nivipes KS7* | *An. nivipes KS7* | Siem Pang, Stung treng, Cambodia | 106°23'N | 14°17'E |
| 8 | *An. nivipes KS8* | *An. nivipes KS8* | *An. nivipes KS8* | Siem Pang, Stung treng, Cambodia | 106°23'N | 14°17'E |
| 9 | *An. nivipes KS9* | *An. nivipes KS9* | *An. nivipes KS9* | Siem Pang, Stung treng, Cambodia | 106°23'N | 14°17'E |
| 10 | *An. nivipes KS10* | *An. nivipes KS10* | *An. nivipes KS10* | Siem Pang, Stung treng, Cambodia | 106°23'N | 14°17'E |
| 11 | *An. nivipes KS11* | *An. nivipes KS11* | *An. nivipes KS11* | Siem Pang, Stung treng, Cambodia | 106°23'N | 14°17'E |
| 12 | *An. nivipes KS12* | *An. nivipes KS12* | *An. nivipes KS12* | Siem Pang, Stung treng, Cambodia | 106°23'N | 14°17'E |
| 13 | *An. nivipes KS13* | *An. nivipes KS13* | *An. nivipes KS13* | Siem Pang, Stung treng, Cambodia | 106°23'N | 14°17'E |
| 14 | *An. nivipes KS14* | *An. nivipes KS14* | *An. nivipes KS14* | Siem Pang, Stung treng, Cambodia | 106°23'N | 14°17'E |
| 15 | *An. nivipes KS15* | *An. nivipes KS15* | *An. nivipes KS15* | Siem Pang, Stung treng, Cambodia | 106°23'N | 14°17'E |
| 16 | *An. nivipes KS16* | *An. nivipes KS16* | *An. nivipes KS16* | Siem Pang, Stung treng, Cambodia | 106°23'N | 14°17'E |
| 17 | *An. nivipes KS17* | *An. nivipes KS17* | *An. nivipes KS17* | Siem Pang, Stung treng, Cambodia | 106°23'N | 14°17'E |
| 18 | *An. nivipes KS19* | *An. nivipes KS19* | *An. nivipes KS19* | Siem Pang, Stung treng, Cambodia | 106°23'N | 14°17'E |
| 19 | *An. nivipes KS20* | *An. nivipes KS20* | *An. nivipes KS20* | Siem Pang, Stung treng, Cambodia | 106°23'N | 14°17'E |
| 20 | *An. nivipes KS21* | *An. nivipes KS21* | *An. nivipes KS21* | Siem Pang, Stung treng, Cambodia | 106°23'N | 14°17'E |
| 21 | *An. nivipes KS22* | *An. nivipes KS22* | *An. nivipes KS22* | Siem Pang, Stung treng, Cambodia | 106°23'N | 14°17'E |
| 22 | *An. nivipes KS23* | *An. nivipes KS23* | *An. nivipes KS23* | Siem Pang, Stung treng, Cambodia | 106°23'N | 14°17'E |
| 23 | *An. nivipes KS24* | *An. nivipes KS24* | *An. nivipes KS24* | Siem Pang, Stung treng, Cambodia | 106°23'N | 14°17'E |
| 24 | *An. nivipes KS25* | *An. nivipes KS25* | *An. nivipes KS25* | Siem Pang, Stung treng, Cambodia | 106°23'N | 14°17'E |
| 25 | *An. nivipes KS26* | *An. nivipes KS26* | *An. nivipes KS26* | Siem Pang, Stung treng, Cambodia | 106°23'N | 14°17'E |
| 26 | *An. nivipes KS27* | *An. nivipes KS27* | *An. nivipes KS27* | Siem Pang, Stung treng, Cambodia | 106°23'N | 14°17'E |
| 27 | *An. nivipes KS28* | *An. nivipes KS28* | *An. nivipes KS28* | Siem Pang, Stung treng, Cambodia | 106°23'N | 14°17'E |
| 28 | *An. nivipes KS29* | *An. nivipes KS29* | *An. nivipes KS29* | Siem Pang, Stung treng, Cambodia | 106°23'N | 14°17'E |
| 29 | *An. nivipes KS30* | *An. nivipes KS30* | *An. nivipes KS30* | Siem Pang, Stung treng, Cambodia | 106°23'N | 14°17'E |
| 30 | *An. nivipes KS31* | *An. nivipes KS31* | *An. nivipes KS31* | Siem Pang, Stung treng, Cambodia | 106°23'N | 14°17'E |
| 31 | *An. nivipes KS32* | *An. nivipes KS32* | *An. nivipes KS32* | Siem Pang, Stung treng, Cambodia | 106°23'N | 14°17'E |
| 32 | *An. nivipes KS33* | *An. nivipes KS33* | *An. nivipes KS33* | Siem Pang, Stung treng, Cambodia | 106°23'N | 14°17'E |
| 33 | *An. nivipes KS34* | *An. nivipes KS34* | *An. nivipes KS34* | Siem Pang, Stung treng, Cambodia | 106°23'N | 14°17'E |
| 34 | *An. nivipes KS35* | *An. nivipes KS35* | *An. nivipes KS35* | Siem Pang, Stung treng, Cambodia | 106°23'N | 14°17'E |
| 35 | *An. nivipes KS36* | *An. nivipes KS36* | *An. nivipes KS36* | Siem Pang, Stung treng, Cambodia | 106°23'N | 14°17'E |
| 36 | *An. nivipes KS37* | *An. nivipes KS37* | *An. nivipes KS37* | Siem Pang, Stung treng, Cambodia | 106°23'N | 14°17'E |
| 37 | *An. nivipes KS38* | *An. nivipes KS38* | */* | Siem Pang, Stung treng, Cambodia | 106°23'N | 14°17'E |
| 38 | *An. nivipes KS39* | *An. nivipes KS39* | *An. nivipes KS39* | Siem Pang, Stung treng, Cambodia | 106°23'N | 14°17'E |
| 39 | *An. nivipes KS40* | *An. nivipes KS40* | */* | Siem Pang, Stung treng, Cambodia | 106°23'N | 14°17'E |
| 40 | *An. nivipes KS41* | *An. nivipes KS41* | *An. nivipes KS41* | Siem Pang, Stung treng, Cambodia | 106°23'N | 14°17'E |
| 41 | *An. nivipes KS43* | *An. nivipes KS43* | *An. nivipes KS43* | Siem Pang, Stung treng, Cambodia | 106°23'N | 14°17'E |
| 42 | *An. nivipes KS44* | *An. nivipes KS44* | *An. nivipes KS44* | Siem Pang, Stung treng, Cambodia | 106°23'N | 14°17'E |
| 43 | *An. nivipes KS45* | *An. nivipes KS45* | *An. nivipes KS45* | Siem Pang, Stung treng, Cambodia | 106°23'N | 14°17'E |
| 44 | *An. nivipes KS46* | *An. nivipes KS46* | */* | Siem Pang, Stung treng, Cambodia | 106°23'N | 14°17'E |
| 45 | *An. nivipes KS47* | *An. nivipes KS47* | *An. nivipes KS47* | Siem Pang, Stung treng, Cambodia | 106°23'N | 14°17'E |
| 46 | *An. nivipes KS48* | *An. nivipes KS48* | *An. nivipes KS48* | Siem Pang, Stung treng, Cambodia | 106°23'N | 14°17'E |
| 47 | *An. nivipes KS49* | *An. nivipes KS49* | *An. nivipes KS49* | Siem Pang, Stung treng, Cambodia | 106°23'N | 14°17'E |
| 48 | *An. nivipes KS50* | *An. nivipes KS50* | *An. nivipes KS50* | Siem Pang, Stung treng, Cambodia | 106°23'N | 14°17'E |
| 49 | *An. nivipes KS51* | *An. nivipes KS51* | */* | Siem Pang, Stung treng, Cambodia | 106°23'N | 14°17'E |
| 50 | *An. nivipes KS52* | *An. nivipes KS52* | *An. nivipes KS52* | Siem Pang, Stung treng, Cambodia | 106°23'N | 14°17'E |
| 51 | *An. nivipes KS53* | *An. nivipes KS53* | *An. nivipes KS53* | Siem Pang, Stung treng, Cambodia | 106°23'N | 14°17'E |
| 52 | *An. nivipes KS54* | *An. nivipes KS54* | *An. nivipes KS54* | Siem Pang, Stung treng, Cambodia | 106°23'N | 14°17'E |
| 53 | *An. nivipes KS55* | *An. nivipes KS55* | */* | Siem Pang, Stung treng, Cambodia | 106°23'N | 14°17'E |
| 54 | *An. nivipes KS56* | *An. nivipes KS56* | *An. nivipes KS56* | Siem Pang, Stung treng, Cambodia | 106°23'N | 14°17'E |
| 55 | *An. nivipes KS57* | *An. nivipes KS57* | *An. nivipes KS57* | Siem Pang, Stung treng, Cambodia | 106°23'N | 14°17'E |
| 56 | *An. nivipes KS58* | *An. nivipes KS58* | *An. nivipes KS58* | Siem Pang, Stung treng, Cambodia | 106°23'N | 14°17'E |
| 57 | *An. nivipes KS60* | *An. nivipes KS60* | *An. nivipes KS60* | Siem Pang, Stung treng, Cambodia | 106°23'N | 14°17'E |
| 58 | *An. nivipes KS61* | *An. nivipes KS61* | *An. nivipes KS61* | Siem Pang, Stung treng, Cambodia | 106°23'N | 14°17'E |
| 59 | *An. nivipes KS62* | *An. nivipes KS62* | *An. nivipes KS62* | Siem Pang, Stung treng, Cambodia | 106°23'N | 14°17'E |

*KS,* Siem Pang County (Stung treng, Cambodia); /, not identified.
