# Supplementary material for: Molecular phylogeny of Anopheles nivipes based on mtDNA-COII and mosquito diversity in Cambodia-Laos border
Source: Malar J. 2022 Mar 17;21:91. doi: 10.1186/s12936-022-04121-w (PMC8932176; doi:10.1186/s12936-022-04121-w)
Supplement: Supplementary file 2 — Additional file 2: Table S2. Genus/Species compositions of mosquitoes trapped by CDC lamp in SIEM PANG County, Stung treng Province. Adult mosquitoes were collected by using overnight trapping with the battery-operated CDC light traps. ** indicated the genus or species composition of mosquitoes which were described in percentage. (%)** indicated the genus or species composition of mosquitoes which were described in numbers. [file 12936_2022_4121_MOESM2_ESM.docx]

**Table S2. Genus/Species compositions of mosquitoes trapped by CDC lamp in SIEM PANG County, Stung treng Province.**

|  | **Genus** | **Species** | **No. collected** | | **Total** |
| --- | --- | --- | --- | --- | --- |
|  |  |  | **Cattle/pig pens** | **Human rooms** |  |
| Mosquito composition** | *Anopheles* | *An. nivipes* | 308 | 23 | 331 |
|  |  | *An. peditaeniatu* | 1 | 0 | 1 |
|  |  | *An. argyropus* | 1 | 0 | 1 |
|  |  | *An. tessellatus* | 4 | 2 | 6 |
|  |  | *An. barbirostris* | 5 | 0 | 5 |
|  |  | *An. sandaicus* | 1 | 1 | 2 |
|  |  | *An. maculatus* | 49 | 15 | 64 |
|  |  | *An. vagus* | 7 | 2 | 9 |
|  |  | *An. interruptus* | 1 | 0 | 1 |
|  |  | *An. dirus* | 0 | 6 | 6 |
|  |  | *An. kochi* | 16 | 2 | 18 |
|  |  | *An. sinensis* | 5 | 1 | 6 |
|  |  | **Total** | **398** | **52** | **450** |
|  | *Culex* | *Cx. tritaeniorhynchus* | 299 | 187 | 486 |
|  |  | *Cx. bitaeniorhynchus* | 2 | 0 | 2 |
|  |  | *Cx. infula* | 1 | 0 | 1 |
|  |  | *Cx. fuscocephala* | 163 | 2 | 165 |
|  |  | *Cx. quinquefasciatus* | 0 | 4 | 4 |
|  |  | *Cx. pseudovishnui* | 27 | 8 | 35 |
|  |  | *Cx. nigropunctatus* | 15 | 3 | 18 |
|  |  | *Cx. halifaxia* | 0 | 1 | 1 |
|  |  | *Cx. gelidus* | 145 | 8 | 153 |
|  |  | **Total** | **652** | **213** | **865** |
|  | *Aedes* | *Ae. lineatopennis* | 11 | 0 | 11 |
|  |  | *Ae. vexans* | 35 | 4 | 39 |
|  |  | *Ae. aegypti* | 0 | 1 | 1 |
|  |  | *Ae. albopictus* | 0 | 1 | 1 |
|  |  | **Total** | **46** | **6** | **52** |
|  | *Armigeres* | *Ar. Subalbatus* | 28 | 11 | 39 |
|  |  | **Total** | **28** | **11** | **39** |
|  | *Tripteroides* | *Tr. Pallidothorax* | 0 | 1 | 1 |
|  |  | **Total** | **0** | **1** | **1** |
|  | Unspecified | **Total** | **15** | **18** | **33** |
|  | **Total collected** | | **1139** | **301** | **1440** |

|  | **Genus** | **Species** | **No. collected** | | **Total** |
| --- | --- | --- | --- | --- | --- |
|  |  |  | **Cattle/pig pens** | **Human rooms** |  |
| Mosquito composition (%)** | *Anopheles* | *An. nivipes* | 27.04% | 7.64% | 22.99% |
|  |  | *An. peditaeniatu* | 0.09% | 0.00% | 0.07% |
|  |  | *An. argyropus* | 0.09% | 0.00% | 0.07% |
|  |  | *An. tessellatus* | 0.35% | 0.66% | 0.42% |
|  |  | *An. barbirostris* | 0.44% | 0.00% | 0.35% |
|  |  | *An. sandaicus* | 0.09% | 0.33% | 0.14% |
|  |  | *An. maculatus* | 4.30% | 4.98% | 4.44% |
|  |  | *An. vagus* | 0.61% | 0.66% | 0.63% |
|  |  | *An. interruptus* | 0.09% | 0.00% | 0.07% |
|  |  | *An. dirus* | 0.00% | 1.99% | 0.42% |
|  |  | *An. kochi* | 1.40% | 0.66% | 1.25% |
|  |  | *An. sinensis* | 0.44% | 0.33% | 0.42% |
|  |  | **Total** | **34.94%** | **17.28%** | **31.25%** |
|  | *Culex* | *Cx. tritaeniorhynchus* | 26.25% | 62.13% | 33.75% |
|  |  | *Cx. bitaeniorhynchus* | 0.18% | 0.00% | 0.14% |
|  |  | *Cx. infula* | 0.09% | 0.00% | 0.07% |
|  |  | *Cx. fuscocephala* | 14.31% | 0.66% | 11.46% |
|  |  | *Cx. quinquefasciatus* | 0.00% | 1.33% | 0.28% |
|  |  | *Cx. pseudovishnui* | 2.37% | 2.66% | 2.43% |
|  |  | *Cx. nigropunctatus* | 1.32% | 1.00% | 1.25% |
|  |  | *Cx. halifaxia* | 0.00% | 0.33% | 0.07% |
|  |  | *Cx. gelidus* | 12.73% | 2.66% | 10.63% |
|  |  | **Total** | **57.24%** | **70.76%** | **60.07%** |
|  | *Aedes* | *Ae. lineatopennis* | 0.97% | 0.00% | 0.76% |
|  |  | *Ae. vexans* | 3.07% | 1.33% | 2.71% |
|  |  | *Ae. aegypti* | 0.00% | 0.33% | 0.07% |
|  |  | *Ae. albopictus* | 0.00% | 0.33% | 0.07% |
|  |  | **Total** | **4.04%** | **1.99%** | **3.61%** |
|  | *Armigeres* | *Ar. Subalbatus* | 2.46% | 3.65% | 2.71% |
|  |  | **Total** | **2.46%** | **3.65%** | **2.71%** |
|  | *Tripteroides* | *Tr. Pallidothorax* | 0.00% | 0.33% | 0.07% |
|  |  | **Total** | **0.00%** | **0.33%** | **0.07%** |
|  | Unspecified | **Total** | **1.32%** | **5.98%** | **2.29%** |
|  | **Total collected** | | **1139** | **301** | **1440** |

Adult mosquitoes were collected by using overnight trapping with the battery-operated CDC light traps. ^**^ indicated the genus or species composition of mosquitoes which were described in percentage. (%)^**^ indicated the genus or species composition of mosquitoes which were described in numbers.
