# Supplementary material for: Molecular phylogeny of Anopheles nivipes based on mtDNA-COII and mosquito diversity in Cambodia-Laos border
Source: Malar J. 2022 Mar 17;21:91. doi: 10.1186/s12936-022-04121-w (PMC8932176; doi:10.1186/s12936-022-04121-w)
Supplement: Supplementary file 3 — Additional file 3: Table S3. COII sequences of Anopheles nivipes were downloaded from the NCBI. * indicated the longitude and latitude coordinates to the geographical center of a certain province, due to the samples being initially collected from various sampling sites in a certain province. An. niv, Anopheles nivipes. [file 12936_2022_4121_MOESM3_ESM.docx]

**Table S3. COII sequences of *Anopheles nivipes* downloaded from the NCBI**

| **ID** | **No.** | **Location** | **Author** | **Longitude** | **Latitude** |
| --- | --- | --- | --- | --- | --- |
| FJ526476.1 | *An. niv* (MM-1) | Myanmar | Morgan,K., et al. | 97.480 | 25.720 |
| FJ526475.1 | *An. niv* (MM-2) | Myanmar | Morgan,K., et al. | 97.480 | 25.720 |
| FJ526474.1 | *An. niv* (TH-1) | Thailand | Morgan,K., et al. | 99.000 | 19.200 |
| FJ526473.1 | *An. niv* (TH-2) | Thailand | Morgan,K., et al. | 99.000 | 19.200 |
| KX148468.1 | *An. niv* (IN-Pun-Ba1) | India: Bathinda, Punjab | Singh,D. and Vashist,D. | 75.229 | 30.272 |
| KX096877.1 | *An. niv* (IN-Pun-Ba2) | India: Bathinda, Punjab | Vashist,D. and Singh,D. | 75.229 | 30.272 |
| KX148464.1 | *An. niv* (IN-Pun-Ch1) | India: Cheema, Punjab | Vashist,D. and Singh,D. | 75.749 | 30.125 |
| KX148463.1 | *An. niv* (IN-Pun-Ch2) | India: Cheema, Punjab | Vashist,D. and Singh,D. | 75.749 | 30.125 |
| KM025238.1 | *An. niv* (IN-Tri1) | India: Tripura | Sarma,N.P., et al. | 91.740^*^ | 23.743^*^ |
| KM025237.1 | *An. niv* (IN-Tri2) | India: Tripura | Sarma,N.P., et al. | 91.740^*^ | 23.743^*^ |
| KM025236.1 | *An. niv* (IN-Tri3) | India: Tripura | Sarma,N.P., et al. | 91.740^*^ | 23.743^*^ |
| KM025235.1 | *An. niv* (IN-Tri4) | India: Tripura | Sarma,N.P., et al. | 91.740^*^ | 23.743^*^ |
| KM025234.1 | *An. niv* (IN-Meg1) | India: Meghalaya | Sarma,N.P., et al. | 90.564^*^ | 25.537^*^ |
| KM025233.1 | *An. niv* (IN-Meg2) | India: Meghalaya | Sarma,N.P., et al. | 90.564^*^ | 25.537^*^ |
| KM025232.1 | *An. niv* (IN-Meg3) | India: Meghalaya | Sarma,N.P., et al. | 90.564^*^ | 25.537^*^ |
| KM025231.1 | *An. niv* (IN-Meg4) | India: Meghalaya | Sarma,N.P., et al. | 90.564^*^ | 25.537^*^ |
| KM025230.1 | *An. niv* (IN-Meg5) | India: Meghalaya | Sarma,N.P., et al. | 90.564^*^ | 25.537^*^ |
| KM025229.1 | *An. niv* (IN-Meg6) | India: Meghalaya | Sarma,N.P., et al. | 90.564^*^ | 25.537^*^ |
| KM025228.1 | *An. niv* (IN-Ass1) | India: Assam | Sarma,N.P., et al. | 93.350^*^ | 26.547^*^ |
| KM025227.1 | *An. niv* (IN-Ass2) | India: Assam | Sarma,N.P., et al. | 93.350^*^ | 26.547^*^ |
| KM025226.1 | *An. niv* (IN-Ass3) | India: Assam | Sarma,N.P., et al. | 93.350^*^ | 26.547^*^ |
| KM025212.1 | *An. niv* (IN-Ass4) | India: Assam | Sarma,N.P., et al. | 93.350^*^ | 26.547^*^ |
| KM025211.1 | *An. niv* (IN-Ass5) | India: Assam | Sarma,N.P., et al. | 93.350^*^ | 26.547^*^ |
| KM025210.1 | *An. niv* (IN-Ass6) | India: Assam | Sarma,N.P., et al. | 93.350^*^ | 26.547^*^ |
| KM025209.1 | *An. niv* (IN-Ass7) | India: Assam | Sarma,N.P., et al. | 93.350^*^ | 26.547^*^ |
| KM025225.1 | *An. niv* (IN-Nag1) | India: Nagaland | Sarma,N.P., et al. | 94.453^*^ | 26.045^*^ |
| KM025224.1 | *An. niv* (IN-Nag2) | India: Nagaland | Sarma,N.P., et al. | 94.453^*^ | 26.045^*^ |
| KM025223.1 | *An. niv* (IN-Nag3) | India: Nagaland | Sarma,N.P., et al. | 94.453^*^ | 26.045^*^ |
| KM025222.1 | *An. niv* (IN-Nag4) | India: Nagaland | Sarma,N.P., et al. | 94.453^*^ | 26.045^*^ |
| KM025221.1 | *An. niv* (IN-Nag5) | India: Nagaland | Sarma,N.P., et al. | 94.453^*^ | 26.045^*^ |
| KM025220.1 | *An. niv* (IN-Nag6) | India: Nagaland | Sarma,N.P., et al. | 94.453^*^ | 26.045^*^ |
| KM025214.1 | *An. niv* (IN-Miz1) | India: Mizoram | Sarma,N.P., et al. | 92.834^*^ | 23.298^*^ |
| KM025213.1 | *An. niv* (IN-Miz2) | India: Mizoram | Sarma,N.P., et al. | 92.834^*^ | 23.298^*^ |

* indicated the longitude and latitude coordinates to the geographical center of a certain province, due to the samples were initially collected from various sampling sites in a certain province. *An. niv*, *Anopheles nivipes.*
