# Supplementary figures and images for: Molecular phylogeny of Anopheles nivipes based on mtDNA-COII and mosquito diversity in Cambodia-Laos border
Source: Malar J. 2022 Mar 17;21:91. doi: 10.1186/s12936-022-04121-w (PMC8932176; doi:10.1186/s12936-022-04121-w)

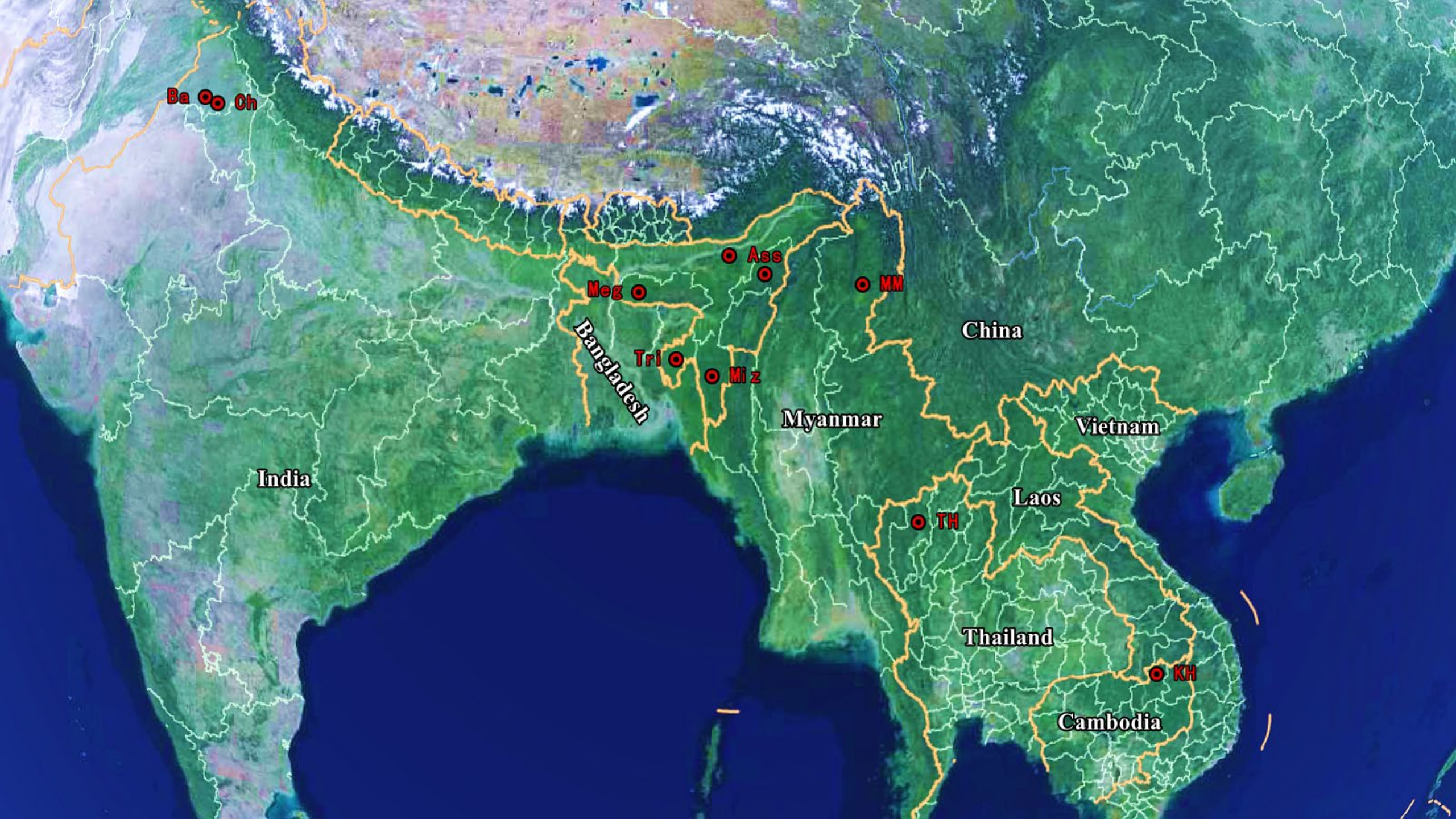

Ba Oh

Ass

MM

Meg

Tri

Miz

China

Myanmar

Vietnam

India

Bangladesh

Laos

TH

Thailand

KH

Cambodia

Supplement: Supplementary file 4 — Additional file 4: Figure S1. Map of the populations from different geographical regions. Populations of mitochondrial COII sequences: KH, Siem Pang County (Stung treng, Cambodia); Tri, Tripura (India); Ch, Cheema (Punjab, India); Ba, Bathinda (Punjab, India); Nag, Nagaland (India); Ass, Assam (India); MM, Myanmar; TH, Thailand; Miz, Mizoram (India); Meg, Meghalaya (India). The map was prepared by using LocaSpace Viewer. [file 12936_2022_4121_MOESM4_ESM.pdf]

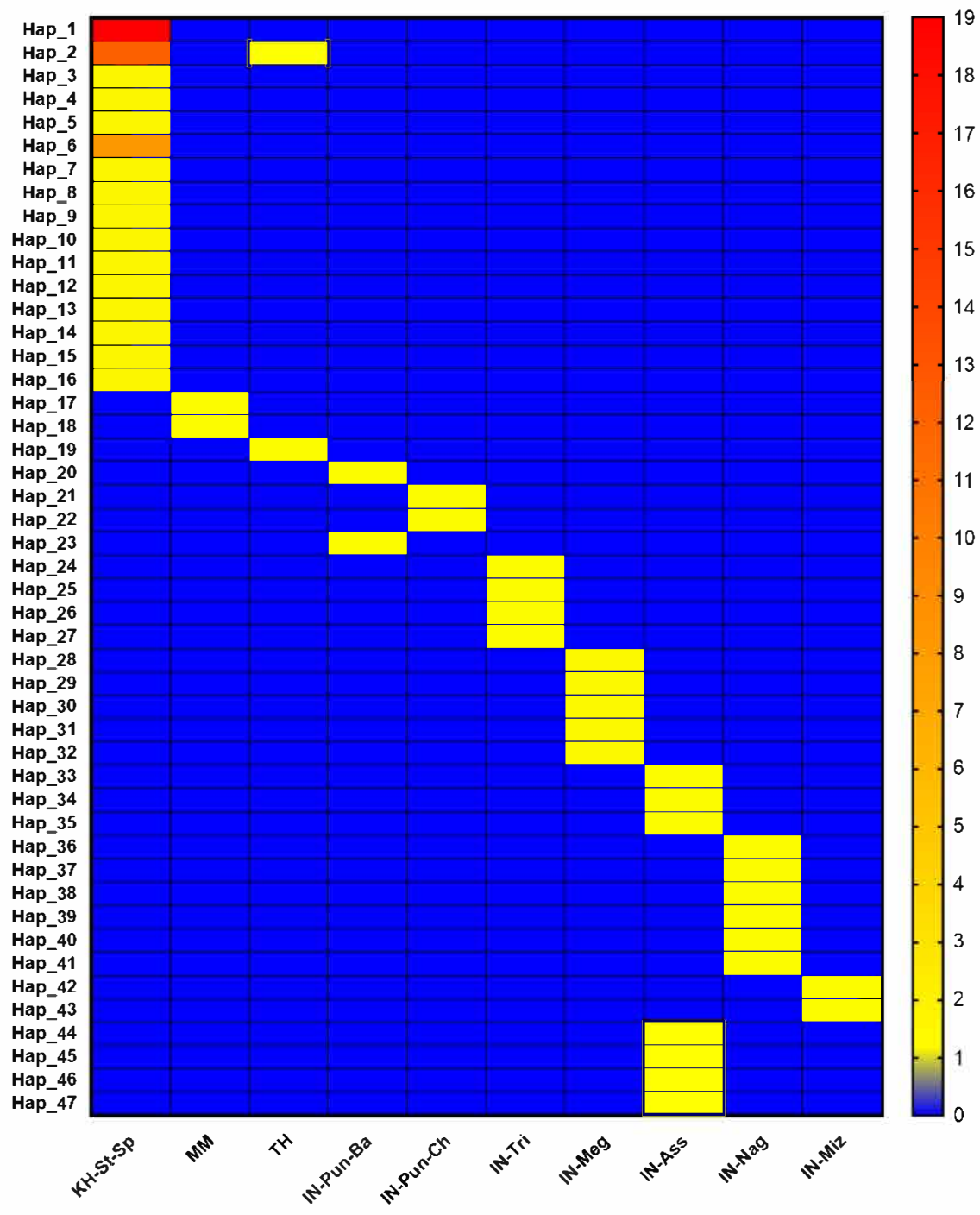

Supplement: Supplementary file 5 — Additional file 5: Figure S2. Distribution heatmap of haplotype based on COII. KH-St-Sp, Siem Pang County (Stung treng, Cambodia); MM, Myanmar; TH, Thailand; IN-Pun-Ba, Bathinda (Punjab, India); IN-Pun-Ch, Cheema (Punjab, India); IN-Tri, Tripura (India); IN-Meg, Meghalaya (India); IN-Ass, Assam (India); IN-Nag, Nagaland (India); IN-Miz, Mizoram (India). The numbers of haplotypes are shown on the right side of the figure. The color scale ranges from blue to red, showing a range from minimum number (0) to maximum numbers (19) for each haplotype. [file 12936_2022_4121_MOESM5_ESM.pdf]

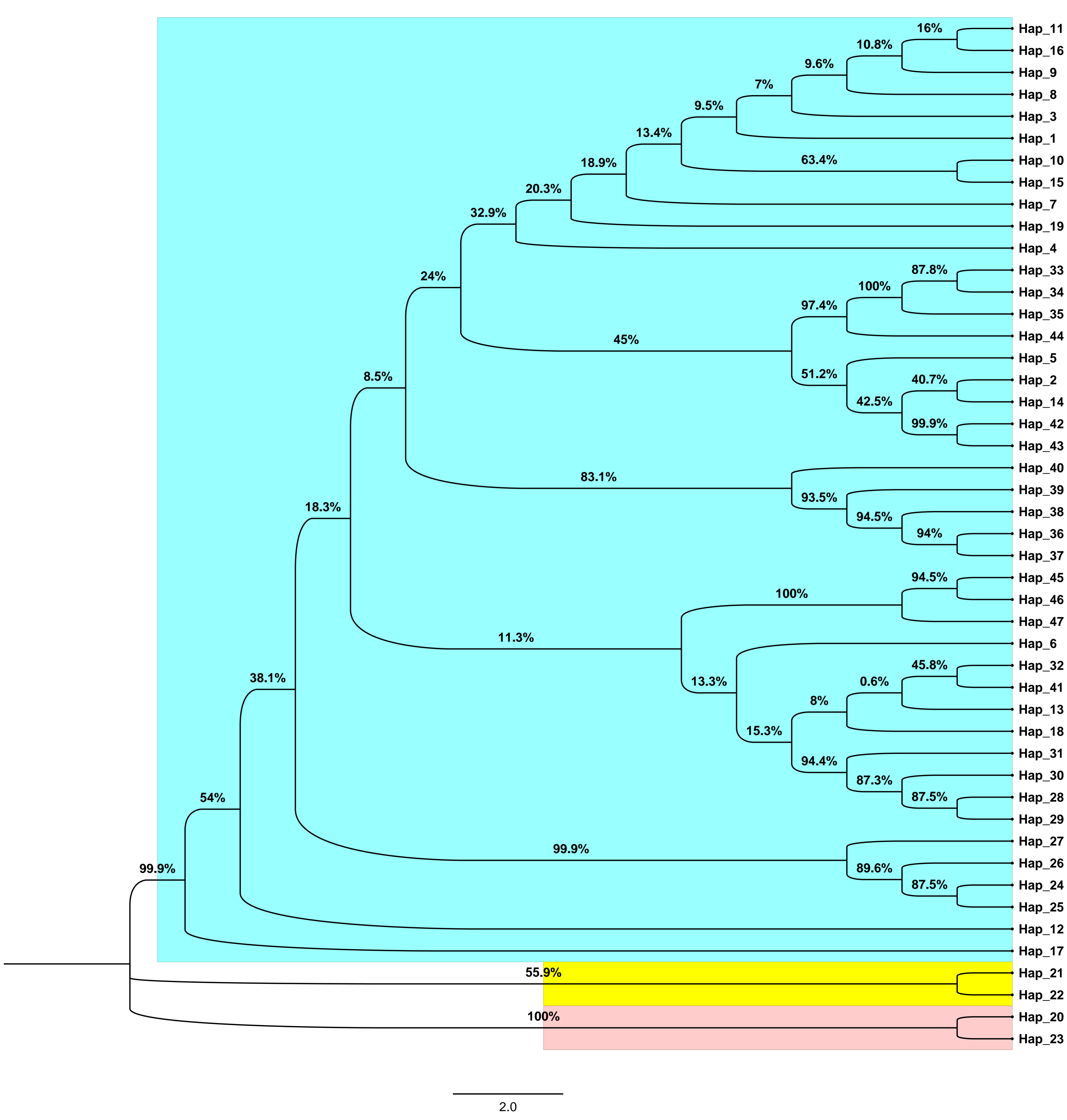

Supplement: Supplementary file 6 — Additional file 6: Figure S3. Neighbor-joining phylogenetic tree of An. nivipes haplotypes based on COII sequences from GenBank and original data in this study. Bootstrap values (1000 replicates) of Neighbor-Joining analyses are shown above/below the main lineages. Lineage designation is indicated on the right. Bars represent 2.0 substitutions per site based on COII. Different colors indicated different population groups of An. nivipes. [file 12936_2022_4121_MOESM6_ESM.pdf]

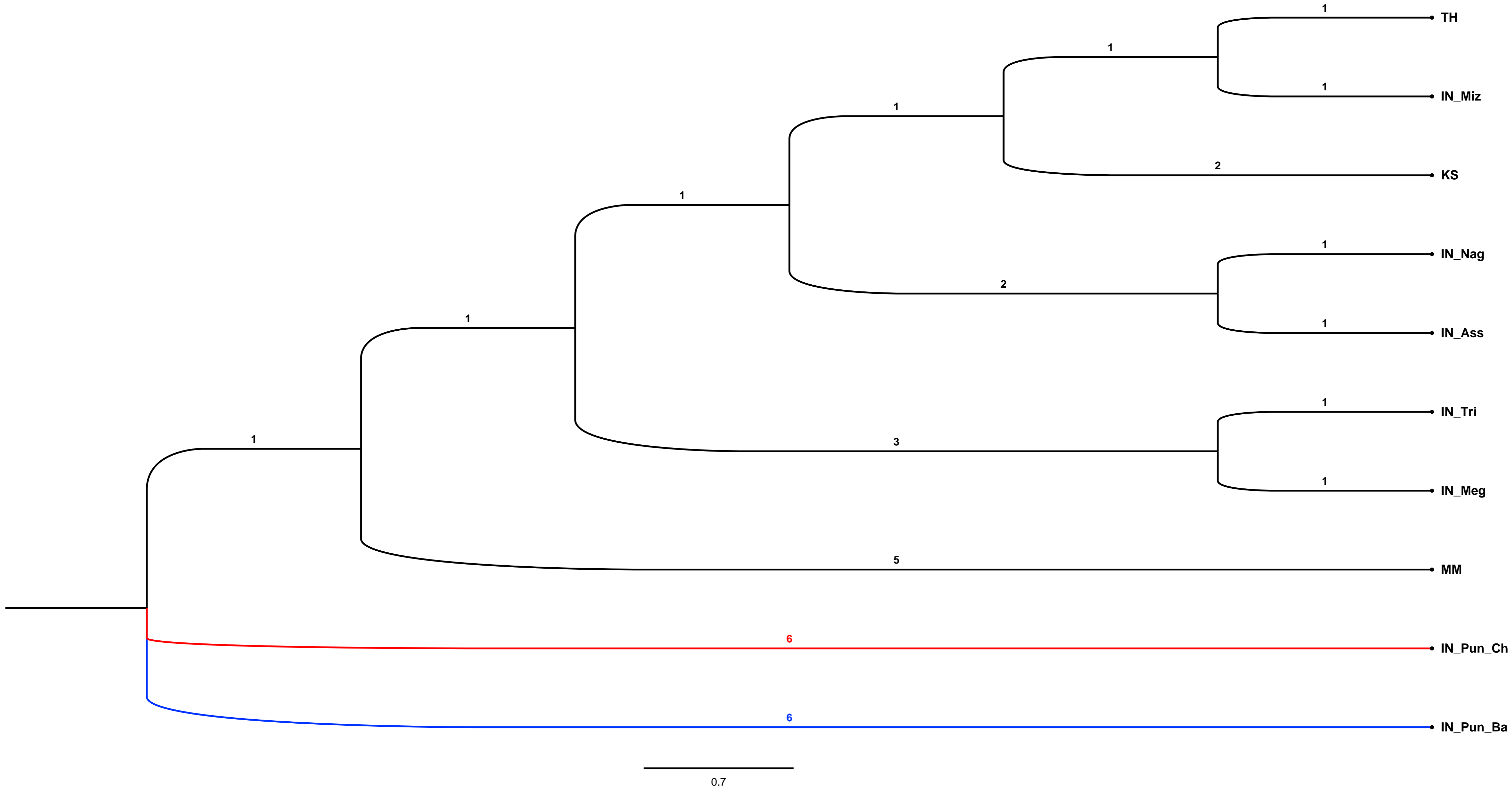

Supplement: Supplementary file 8 — Additional file 8: Figure S4. Cluster analysis based on COII sequences in Anopheles nivipes populations. UPGMA dendrogram based on Nei ‘s unbiased genetic distance between different populations of An. nivipes. Bars represent 0.7 substitutions per site based on COII. KS, Siem Pang County (Stung treng, Cambodia); IN_Tri, Tripura (India); IN_Pun_Ch, Cheema (Punjab, India); IN_Pun_Ba, Bathinda (Punjab, India); IN_Nag, Nagaland (India); IN_Ass, Assam (India); MM, Myanmar; TH, Thailand; IN_Miz, Mizoram (India); IN_Meg, Meghalaya (India). [file 12936_2022_4121_MOESM8_ESM.pdf]

**a**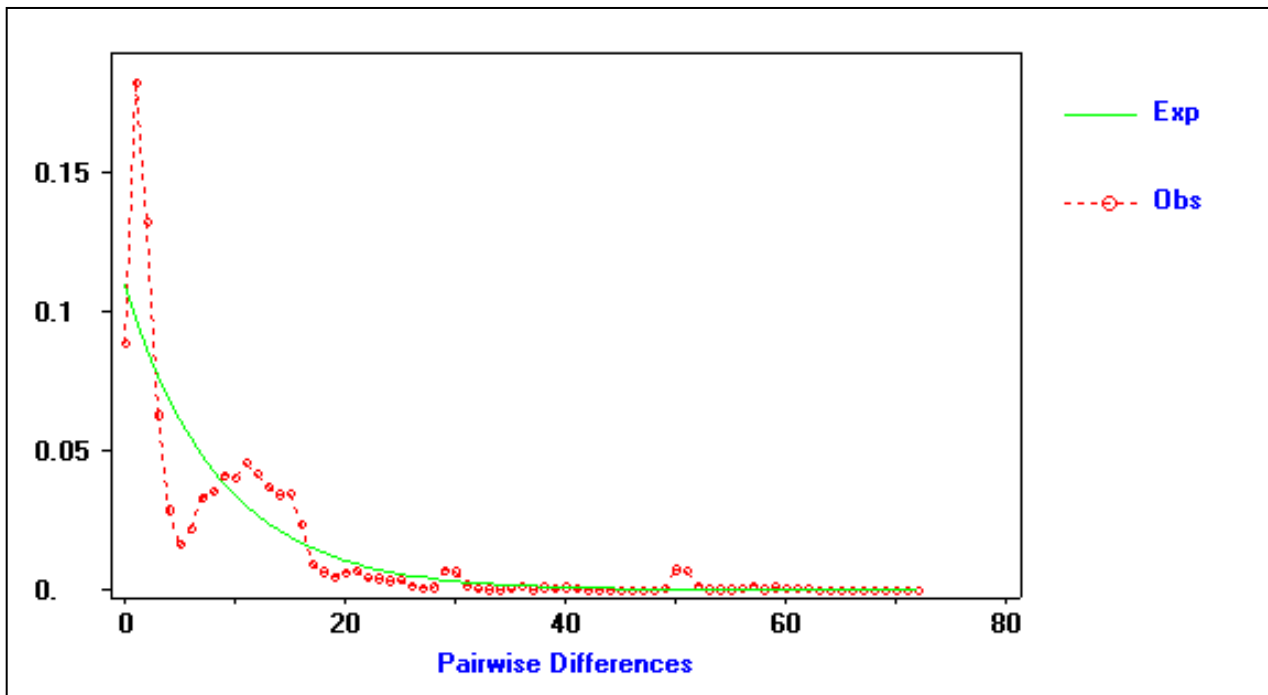**b**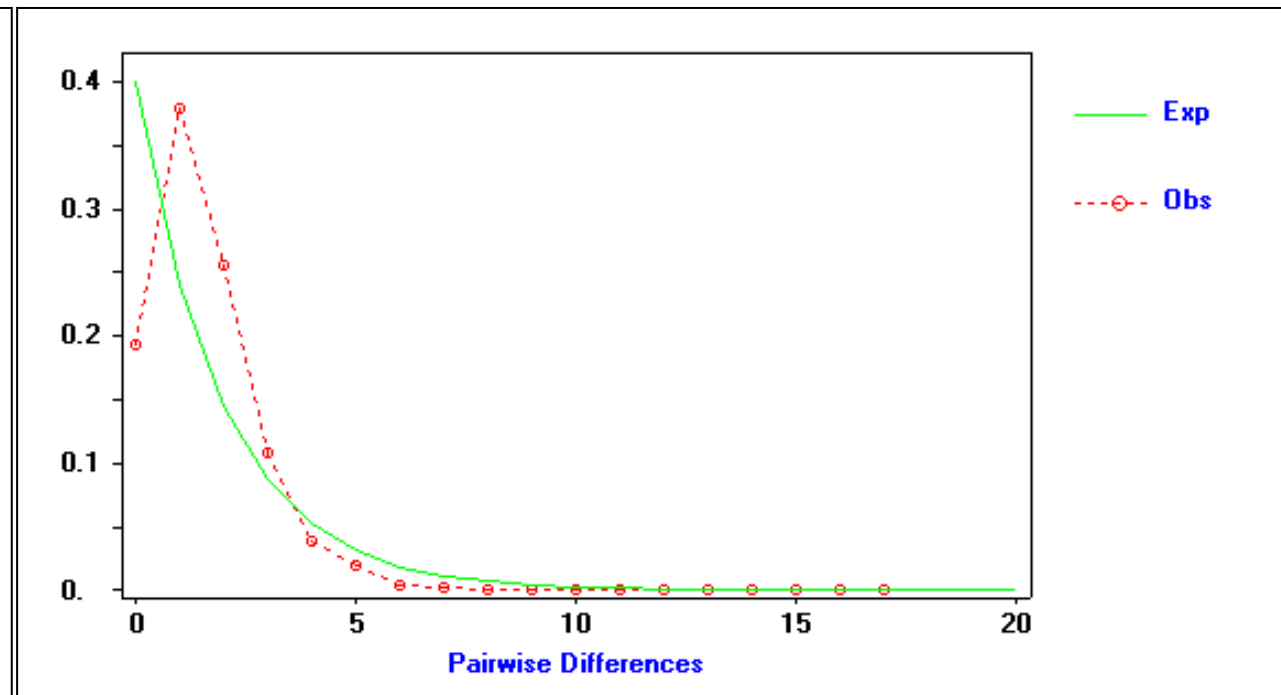

Supplement: Supplementary file 9 — Additional file 9: Figure S5. Mismatch distribution graphs for Siem Pang population. The X and Y-axis show the number of pairwise differences and the frequency of the pairwise comparisons, respectively. The observed frequencies are represented by a dotted line. The frequency expected under the hypothesis of the constant population model is depicted by a solid line. (a) all populations-COII; (b) Siem Pang population-COII. [file 12936_2022_4121_MOESM9_ESM.pdf]
