# Supplementary material for: Molecular phylogeny of Anopheles nivipes based on mtDNA-COII and mosquito diversity in Cambodia-Laos border
Source: Malar J. 2022 Mar 17;21:91. doi: 10.1186/s12936-022-04121-w (PMC8932176; doi:10.1186/s12936-022-04121-w)
Supplement: Supplementary file 7 — Additional file 7: Table S4. Genetic differentiation and Gene flow among the Geographic Groups based on COII. The pairwise FST values and Nm values based on the COII are shown below and above the diagonal, respectively. Characters in bold indicate the significance (P < 0.05). inf, infinite. KH_St_Sp, Siem Pang County (Stung treng, Cambodia); IN_Tri, Tripura (India); IN_Pun_Ch, Cheema (Punjab, India); IN_Pun_Ba, Bathinda (Punjab, India); IN_Nag, Nagaland (India); IN_Ass, Assam (India); MM, Myanmar; TH, Thailand; IN_Miz, Mizoram (India); IN-Meg, Meghalaya (India). [file 12936_2022_4121_MOESM7_ESM.docx]

**Table S4. Genetic differentiation and Gene flow among the Geographic Groups based on COII**

|  | KS_St_Sp | MM | TH | IN_Tri | IN_Meg | IN_Nag | IN_Miz | IN_Ass | IN_Pun_Ba | IN_Pun_Ch |
| --- | --- | --- | --- | --- | --- | --- | --- | --- | --- | --- |
| KS_St_Sp |  | 0.42382 | 3.50176 | 0.08886 | 0.07338 | 0.10438 | 0.11013 | 0.27362 | 0.01365 | 0.01112 |
| MM | **0.54123** |  | 1.66667 | 0.26906 | 0.2149 | 0.45821 | 0.13889 | 8.44874 | 0.20303 | 0.05208 |
| TH | 0.12495 | 0.23077 |  | 0.24615 | 0.19898 | 0.39733 | 0.25 | 4.64458 | 0.20606 | 0.05405 |
| IN_Tri | **0.8491** | 0.65015 | 0.6701 |  | 0.12842 | 0.19893 | 0.12403 | 0.55434 | 0.09793 | 0.03966 |
| IN_Meg | **0.87202** | **0.6994** | **0.71533** | **0.79565** |  | 0.24422 | 0.11471 | 0.37416 | 0.07193 | 0.03666 |
| IN_Nag | **0.8273** | **0.5218** | 0.55721 | **0.71538** | **0.67184** |  | 0.20709 | 0.54799 | 0.08276 | 0.04822 |
| IN_Miz | **0.81949** | 0.78261 | 0.66667 | 0.80124 | **0.8134** | 0.70712 |  | 0.70751 | 0.18994 | 0.04878 |
| IN_Ass | **0.64631** | 0.05587 | 0.09719 | **0.47423** | **0.57198** | **0.4771** | **0.41407** |  | 0.11375 | 0.09307 |
| IN_Pun_Ba | **0.97343** | 0.71121 | 0.70815 | **0.83622** | **0.87423** | **0.85798** | 0.7247 | 0.81467 |  | 1.3 |
| IN_Pun_Ch | **0.97824** | 0.90566 | 0.90244 | 0.9265 | 0.93169 | **0.91203** | 0.91111 | 0.84307 | 0.27778 |  |

The pairwise *F*_ST_ values and Nm values based on the COII are shown below and above the diagonal, respectively. **Characters** in bold indicated the significance (P<0.05). inf, infinite. KH_St_Sp, Siem Pang County (Stung treng, Cambodia); IN_Tri, Tripura (India); IN_Pun_Ch, Cheema (Punjab, India); IN_Pun_Ba, Bathinda (Punjab, India); IN_Nag, Nagaland (India); IN_Ass, Assam (India); MM, Myanmar; TH, Thailand; IN_Miz, Mizoram (India); IN-Meg, Meghalaya (India).
