# Supplementary material for: Molecular phylogeny of Anopheles nivipes based on mtDNA-COII and mosquito diversity in Cambodia-Laos border
Source: Malar J. 2022 Mar 17;21:91. doi: 10.1186/s12936-022-04121-w (PMC8932176; doi:10.1186/s12936-022-04121-w)

**DNA extraction, ITS2 amplification and sequencing**

Genomic DNA was isolated from individual mosquitoes by using the QIAamp® DNA Mini Kit (QIAGEN, Hilden, Germany) following the manufacturer’s instructions. Approximately 356 bp PCR product of the ITS2 region was amplified using primers, ITS2-F (5’-TGTGAACTGCAGGACACAT-3’) and ITS2-R (5’-TATGCTTAAATTCAGGGGGT-3’). ITS2 was amplified with the following cycling parameters: 94 ℃, 2 min; 94 ℃/30 s, 50 ℃/30 s, 72 ℃/40 s for 40 cycles; and a final extension at 72 ℃ for 10 min. The total PCR reaction volume was 20 μl, and the PCR reagent mixture consisted of 2.5 μl of 10 x buffer, 0.2 mM of dNTPs, 0.3 μM of each primer, 0.05 unit of Takara Taq (Dalian, China), and 2 μl of template DNA. The PCR products were analyzed by 1.5% agarose gel electrophoresis stained with GoldView (Solarbio, Beijing, China), under UV transillumination. The sequencing reaction proceeded in both directions using an ABI Big Dye Terminator Kit (Applied Biosystems, Thermo Fisher Scientific). Further analysis was conducted with the assistance of ABI Prism 3500xL Genetic Analyzer (Applied Biosystems, Thermo Fisher Scientific) in Shanghai (Sangon Biotech).

**Table 1. ITS2 sequences of *Anopheles nivipes* downloaded from the NCBI**

| **ID** | **No.** | **Location** | **Author** | **Longitude** | **Latitude** |
| --- | --- | --- | --- | --- | --- |
| JN654431.1 | *An. niv* (IN_Ass_Na1) | India: Assam, Nalbari, Kumarikata | Sarma,N.P. and Prakash,A., et al. | 91.436 | 26.443 |
| JN654430.1 | *An. niv* (IN_Ass_Na2) | India: Assam, Nalbari, Kawali | Sarma,N.P. and Prakash,A., et al. | 91.436 | 26.443 |
| JN654429.1 | *An. niv* (IN_Ass_Na3) | India: Assam, Nalbari, Tamulpur | Sarma,N.P. and Prakash,A., et al. | 91.436 | 26.443 |
| DQ279441.1 | *An. niv* (IN_Ass_Ka1) | India: Assam, Kamrup, Sonapur | Alam,M.T., et al. | 91.969 | 26.112 |
| DQ279440.1 | *An. niv* (IN_Ass_Ka2) | India: Assam, Kamrup, Sonapur | Alam,M.T., et al. | 91.969 | 26.112 |
| DQ279439.1 | *An. niv* (IN_Ass_Ka3) | India: Assam, Kamrup, Sonapur | Alam,M.T., et al. | 91.969 | 26.112 |
| DQ279438.1 | *An. niv* (IN_Ass_Ka4) | India: Assam, Kamrup, Sonapur | Alam,M.T., et al. | 91.969 | 26.112 |
| DQ279437.1 | *An. niv* (IN_Ass_Ka5) | India: Assam, Kamrup, Sonapur | Alam,M.T., et al. | 91.969 | 26.112 |
| DQ279436.1 | *An. niv* (IN_Ass_Ka6) | India: Assam, Kamrup, Sonapur | Alam,M.T., et al. | 91.969 | 26.112 |
| DQ279435.1 | *An. niv* (IN_Ass_Ka7) | India: Assam, Kamrup, Sonapur | Alam,M.T., et al. | 91.969 | 26.112 |
| DQ279434.1 | *An. niv* (IN_Ass_Ka8) | India: Assam, Kamrup, Sonapur | Alam,M.T., et al. | 91.969 | 26.112 |
| DQ013796.1 | *An. niv* (IN_Ass_Ka9) | India: Assam, Kamrup | Alam,M.T., et al. | 91.969 | 26.112 |
| DQ013800.1 | *An. niv* (IN_Ass_Ka10) | India: Assam, Kamrup | Alam,M.T., et al. | 91.969 | 26.112 |
| JQ741975.1 | *An. niv* (IN_Ass_Ka11) | India: Assam, Kamrup | Rabha,B., et al. | 91.969 | 26.112 |
| JN654427.1 | *An. niv* (IN_Miz_Ma1) | India: Mizoram, Mamit, Thenzwal | Sarma,N.P. and Prakash,A., et al. | 92.750 | 23.317 |
| JN643727.1 | *An. niv* (IN_Miz_Ma2) | India: Mizoram, Mamit, Thenzwal | Zomuanpuii,R. and Ringngheti,L., et al. | 92.750 | 23.317 |
| FJ526623.1 | *An. niv* (TH_Cm_Cm1) | Thailand,Chiang Mai | Morgan,K., et al. | 99.000 | 19.200 |
| FJ526622.1 | *An. niv* (TH_Cm_Cm2) | Thailand,Chiang Mai | Morgan,K., et al. | 99.000 | 19.200 |
| JN654426.1 | *An. niv* (IN_Meg_Kh1) | India: Meghalaya, Khasi hills | Sarma,N.P. and Prakash,A., et al. | 91.850 | 26.050 |
| JN654425.1 | *An. niv* (IN_Meg_Kh2) | India: Meghalaya, Khasi hills | Sarma,N.P. and Prakash,A., et al. | 91.850 | 26.050 |

* indicated the longitude and latitude coordinates to the geographical center of a certain province, due to the samples were initially collected from various sampling sites in a certain province. *An. niv*, *Anopheles nivipes.*

**Table 2. Genetic diversity indices and neutrality tests (Fu’s *Fs* and Tajima’s *D*) based on the ITS2 gene of *An. nivipes***

| **Species** | **n** | **Haplotype Code** | **S** | **Pi** | **h** | **Hd** | **k** | **Fu's *Fs*** | **Tajima's *D*** |
| --- | --- | --- | --- | --- | --- | --- | --- | --- | --- |
| Total | 73 | H1(29), H2(31), H3(10), H4(1), H5(1), H6(1) | 9 | 0.00402 | 6 | 0.65100 | 1.39764 | 0.38900 | -0.85972 |
| KH_St_Sp | 53 | H1(29), H2(23), H6(1) | 5 | 0.00193 | 3 | 0.52180 | 0.67271 | 1.07800 | -1.23604 |
| IN_Ass_Na | 3 | H2(3) | 1 | 0.00052 | 2 | 0.18200 | 0.18182 | -0.41000 | -1.12850 |
| IN_Ass_Ka | 11 | H3(11), H4(1) | 0 | 0.00000 | 1 | 0.00000 | 0.00000 | n.d. | n.d. |
| IN_Miz_Ma | 2 | H2(2) | 0 | 0.00000 | 1 | 0.00000 | 0.00000 | n.d. | n.d. |
| IN_Meg_kh | 2 | H2(2) | 3 | 0.00575 | 2 | 0.66670 | 2.00000 | 1.60900 | n.d. |
| TH_Cm | 2 | H2(1), H5(1) | 1 | 0.00287 | 2 | 1.00000 | 1.00000 | 1.00000 | n.d. |

n.d., not determined; n.s., *P* > 0.10; #, *P* < 0.10; ^*^, *P* < 0.05; ^**^, *P* < 0.02; ^***^, *P* < 0.001. Abbreviations: n number of sequences, S number of polymorphic sites, pi nucleotide diversity, h number of haplotypes, Hd haplotype diversity. KH_St_Sp, Siem Pang County (Stung treng, Cambodia); IN_Ass_Na, Nalbari (Assam, India); IN_Ass_Ka, Kamrup (Assam, India); IN_Miz_Ma, Mamit (Mizoram, India); IN_Meg_kh, Khasi hills (Meghalaya, India); TH_Cm, Chiang Mai (Thailand).

**Table 3. Analysis of molecular variance (AMOVA) of six *An. nivipes* populations based on ITS2**

| **Source of variation** | **d. f.** | **Sum of squares** | **Variance components** | **% of variation** | **Fixation index (*P*)** |
| --- | --- | --- | --- | --- | --- |
| Among groups | 1 | 18.242 | 0.08665 Va | 7.120 | *F*_CT_: 0.07116 (*P*<0.001) |
| Among populations within groups | 4 | 13.173 | 0.84895 Vb | 69.720 | *F*_SC_: 0.75060 (*P*<0.001) |
| Within populations | 67 | 18.900 | 0.28208 Vc | 23.170 | *F*_ST_: 0.76834 (*P*>0.05) |
| Total | 72 | 50.315 | 1.21768 |  |  |

*F*_CT_, Fixation index among groups, *F*_SC_, among populations within groups, *F*_ST_, within populations.

**Table 4. Population groups identified by spatial analysis of molecular variance (SAMOVA) algorithm based on ITS2**

| **K** | **Population grouping** | ***F*_CT_** | ***F*_SC_** |
| --- | --- | --- | --- |
| k = 2 | [KH_St_Sp, IN_Ass_Na, IN_Miz_Ma, IN_Meg_Kh, TH_Cm][N_Ass_Ka] | 0.78476^ns^ | 0.27524^ns^ |
| k = 3 | [KH_St_Sp][IN_Ass_Na, IN_Miz_Ma, IN_Meg_Kh, TH_Cm][N_Ass_Ka] | 0.826269^ns^ | -0.32136^ns^ |
| k = 4 | [KH_St_Sp][IN_Ass_Na, IN_Miz_Ma, IN_Meg_Kh][TH_Cm][N_Ass_Ka] | 0.869666^ns^ | -0.77778^ns^ |
| k = 5 | [KH_St_Sp][IN_Ass_Na][IN_Miz_Ma, N_Meg_Kh][TH_Cm][N_Ass_Ka] | 0.883124^ns^ | -1.00000^ns^ |

Significant values ^*^*P*<0.05; ^**^*P*<0.01; ^***^*P*<0.001, ns: Not significant. KH_St_Sp, Siem Pang County (Stung treng, Cambodia); IN_Ass_Na, Nalbari (Assam, India); IN_Ass_Ka, Kamrup (Assam, India); IN_Miz_Ma, Mamit (Mizoram, India); IN_Meg_kh, Khasi hills (Meghalaya, India); TH_Cm, Chiang Mai (Thailand).

**Table 5. Genetic differentiation and Gene flow among the Geographic Groups based on ITS2**

|  | IN_Ass_Na | IN_Meg_Kh | IN_Miz_Ma | IN_Ass_Ka | TH_Cm | KS_St_Sp |
| --- | --- | --- | --- | --- | --- | --- |
| IN_Ass_Na |  | inf | inf | 0.02711 | 1.5 | 1.64065 |
| IN_Meg_Kh | 0.00000 |  | inf | 0.02975 | inf | 2.35274 |
| IN_Miz_Ma | 0.00000 | 0.00000 |  | 0.02975 | inf | 2.35274 |
| IN_Ass_Ka | **0.94857** | **0.94383** | **0.94383** |  | 0.04269 | 0.09557 |
| TH_Cm | 0.25000 | 0.00000 | 0.00000 | **0.92134** |  | 0.88082 |
| KS_St_Sp | 0.23357 | 0.17527 | 0.17527 | **0.83954** | **0.36210** |  |

The pairwise *F*_ST_ values and Nm values based on the ITS2 are shown below and above the diagonal, respectively. **Characters** in bold indicated that the significance (P<0.05). inf, infinite. KH_St_Sp, Siem Pang County (Stung treng, Cambodia); IN_Ass_Na, Nalbari (Assam, India); IN_Ass_Ka, Kamrup (Assam, India); IN_Miz_Ma, Mamit (Mizoram, India); IN_Meg_kh, Khasi hills (Meghalaya, India); TH_Cm, Chiang Mai (Thailand).

**Figure 1.** Map of the populations from different geographical regions. Populations of rDNA ITS2 sequences: KH, Siem Pang County (Stung treng, Cambodia); Na, Nalbari (Assam, India); Ka, Kamrup (Assam, India); Ma, Mamit (Mizoram, India); Kh, Khasi hills (Meghalaya, India); TH, Chiang Mai (Thailand).


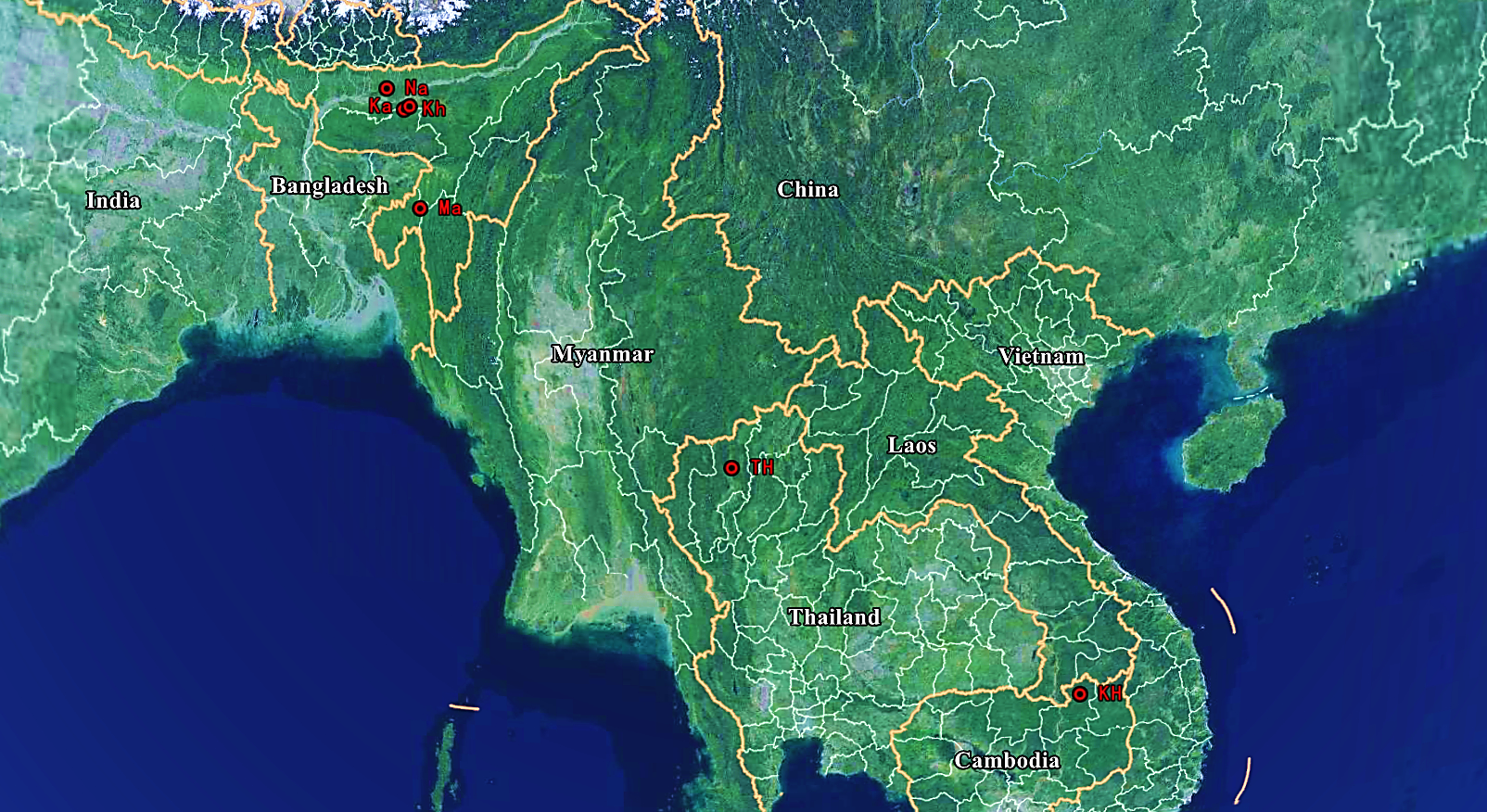


**Figure 2.** Distribution heatmap of haplotype based on ITS2. KH-St-Sp, Siem Pang County (Stung treng, Cambodia); IN-Ass-Na, Nalbari (Assam, India); IN-Ass-Ka, Kamrup (Assam, India); IN-Miz-Ma, Mamit (Mizoram, India); IN-Meg-Kh, Khasi hills (Meghalaya, India); TH-Cm, Chiang Mai (Thailand); The numbers of haplotypes are shown on the right side of the figure. The color scale ranges from blue to red, showing a range from minimum number (0) to maximum numbers (29) for each haplotype.


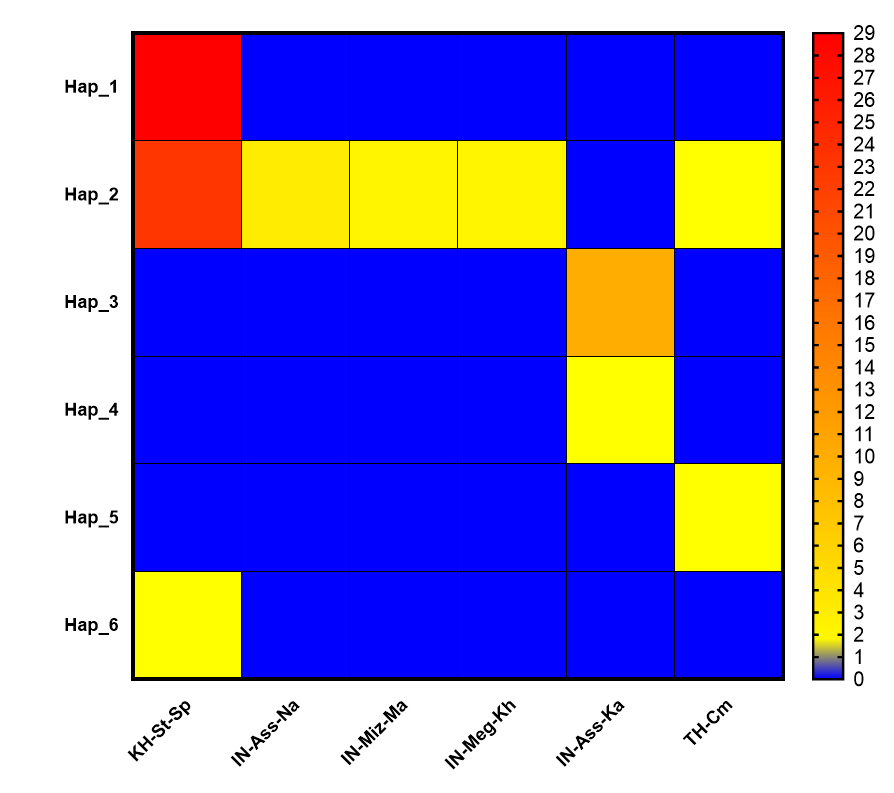


**Figure 3.** Phylogenetic network of 6 rDNA haplotypes of ITS2 gene in *Anopheles nivipes*. Localities are indicated by different colors (bottom-right). The area of each circle is approximately proportional to the frequency of the haplotype. ^#^Samples available in Genbank. ^*^Samples from Cambodia-Laos border. KH_St_Sp, Siem Pang County (Stung treng, Cambodia); IN_Ass_Na, Nalbari (Assam, India); IN_Ass_Ka, Kamrup (Assam, India); IN_Miz_Ma, Mamit (Mizoram, India); IN_Meg_kh, Khasi hills (Meghalaya, India); TH_Cm, Chiang Mai (Thailand).

**
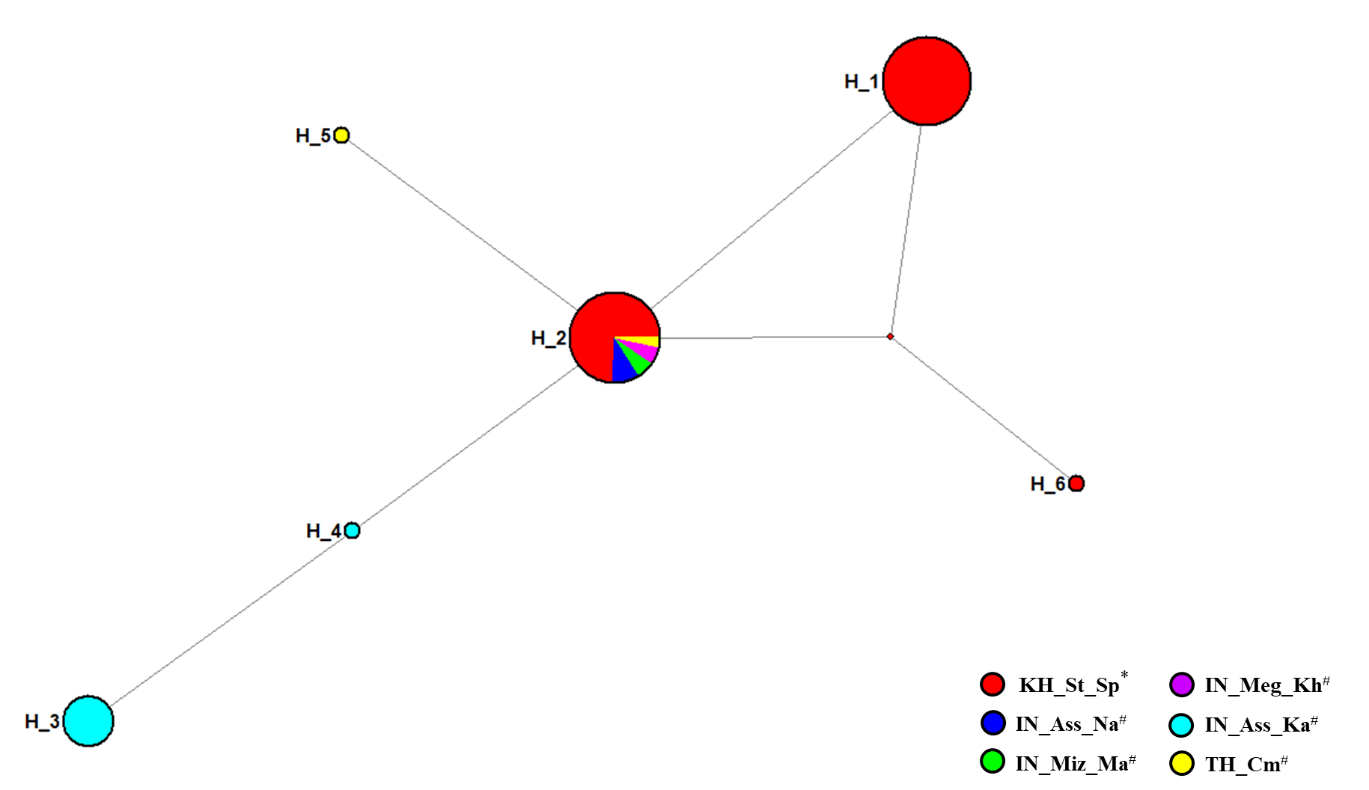
**


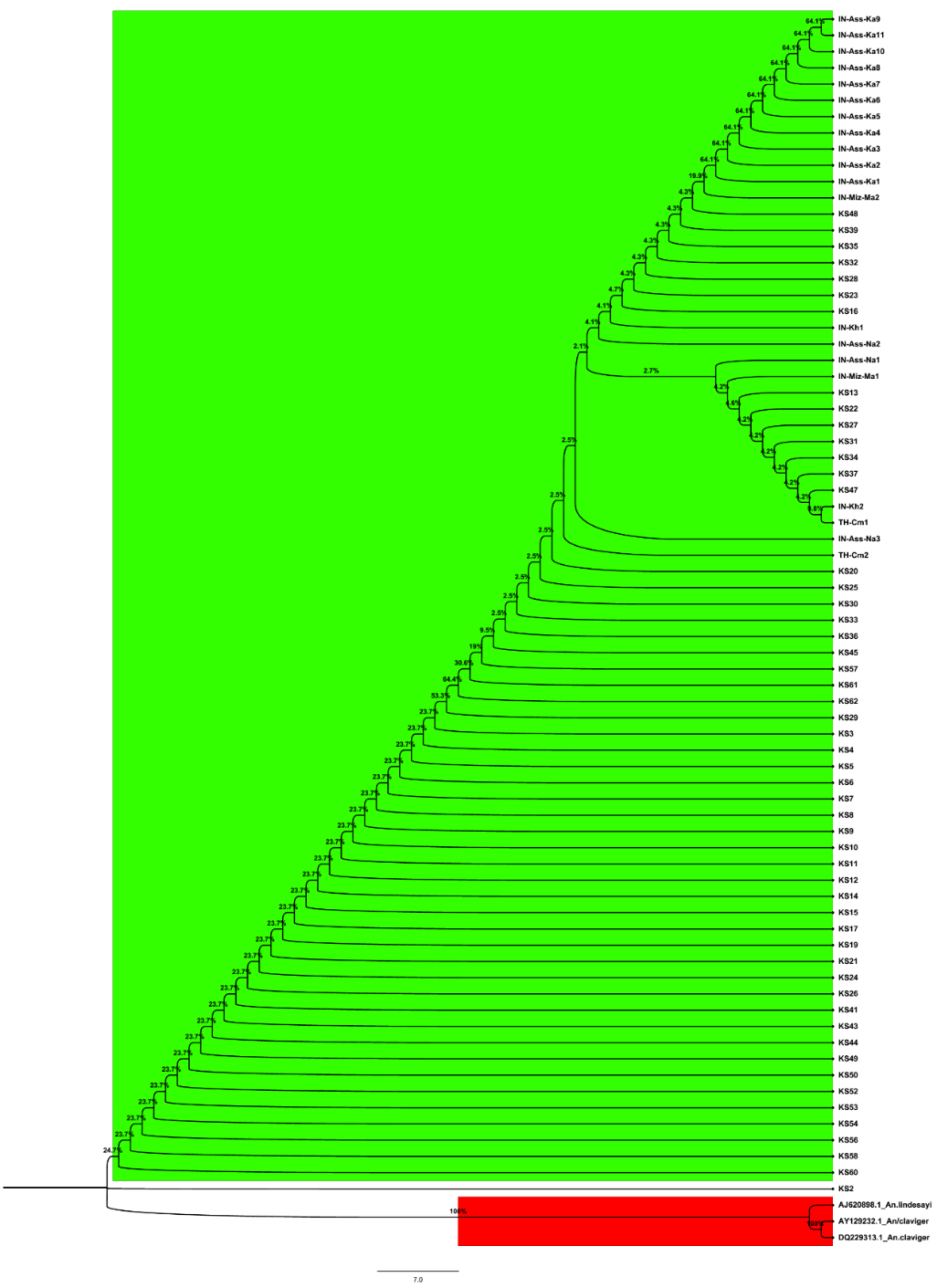
**Figure** **4.** Neighbour-joining phylogenetic tree of *Anopheles nivipes* based on ITS2 from GenBank and our original data. Bootstrap values (1000 replicates) of neighbor-joining analyses are shown above/below the main lineages. Lineage designation is indicated on the right. Bars represent 7.0 substitutions per site based on ITS2. Different colors indicated different population groups of *An. nivipes*. *An. lindesayi* and *An. claviger* was used as the outgroup taxa. KS, Siem Pang County (Stung treng, Cambodia); IN_Ass_Na, Nalbari (Assam, India); IN_Ass_Ka, Kamrup (Assam, India); IN_Miz_Ma, Mamit (Mizoram, India); IN_Meg_Kh, Khasi hills (Meghalaya, India); TH_Cm, Chiang Mai (Thailand).

**Figure 5.** Isolation by distance, the relationship between geographical and genetic distances based on ITS2 sequences in *Anopheles nivipes* populations. Isolation by distance (IBD) was examined using a nonparametric Mantel with the web-based computer program IBDWS v.3.16.


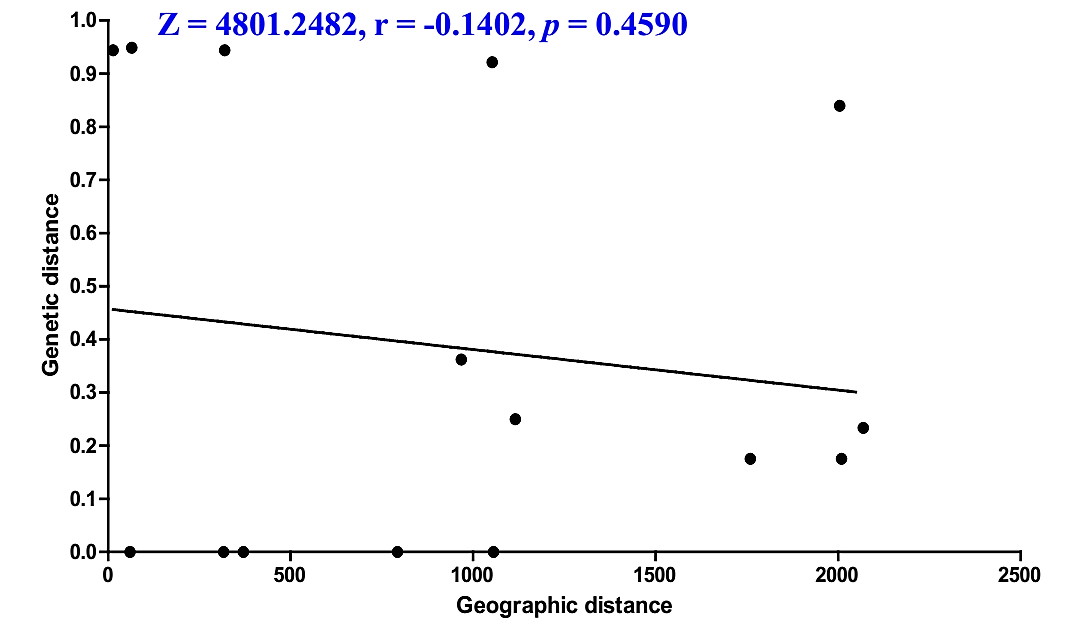


**Figure 6.** Neighbor-joining phylogenetic tree of *An. nivipes* haplotypes based on ITS2 sequences from GenBank and our original data. Bootstrap values (1000 replicates) of Neighbor-Joining analyses are shown above/below the main lineages. Lineage designation is indicated on the right. Bars represent 0.4 substitutions per site based on ITS2. Different colors indicated different population groups of *An. nivipes*.


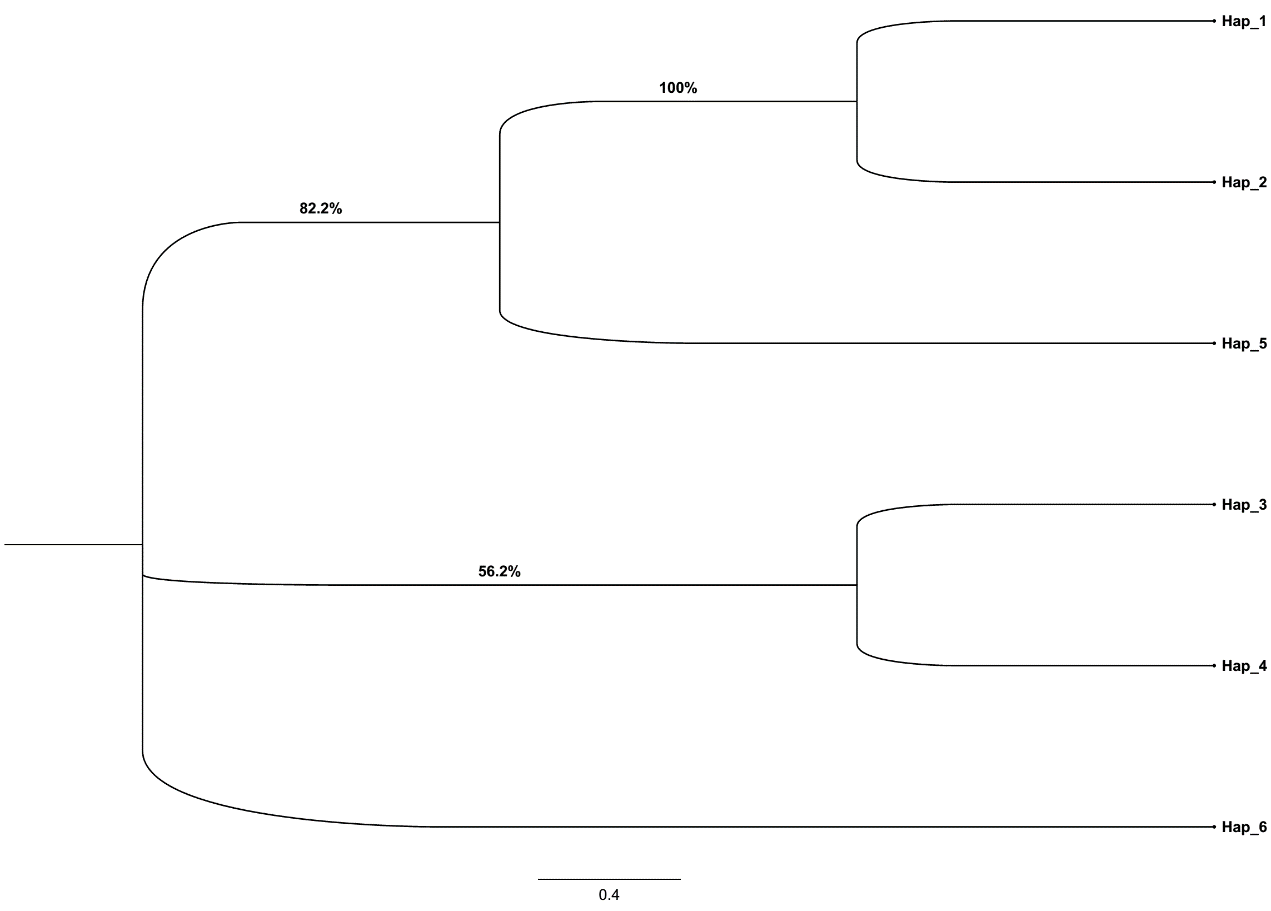


**Figure 7.** Cluster analysis based on ITS2 sequences in *Anopheles nivipes* populations. UPGMA dendrogram based on Nei‘s unbiased genetic distance between different populations of *An. nivipes*. Bars represent 0.5 substitutions per site based on ITS2. KS, Siem Pang County (Stung treng, Cambodia); IN_Ass_Na, Nalbari (Assam, India); IN_Ass_Ka, Kamrup (Assam, India); IN_Miz_Ma, Mamit (Mizoram, India); IN_Meg_Kh, Khasi hills (Meghalaya, India); TH_Cm_Cm, Chiang Mai (Thailand).


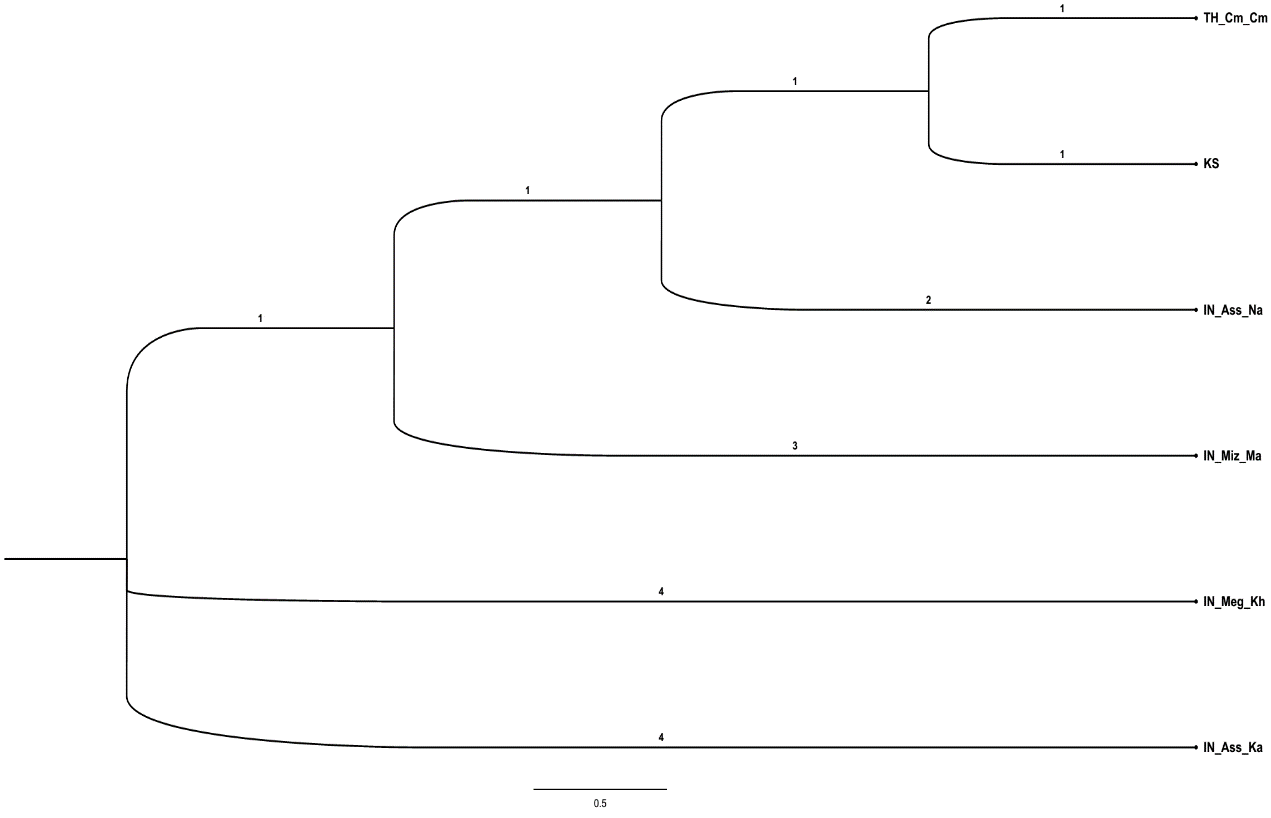


**Figure 8.** Mismatch distribution graphs for Siem Pang population. The X and Y-axis show the number of pairwise differences and the frequency of the pairwise comparisons, respectively. The observed frequencies are represented by a dotted line. The frequency expected under the hypothesis of constant population model is depicted by a solid line. **(a)** all populations-ITS2; (**b)** Siem Pang population-ITS2.


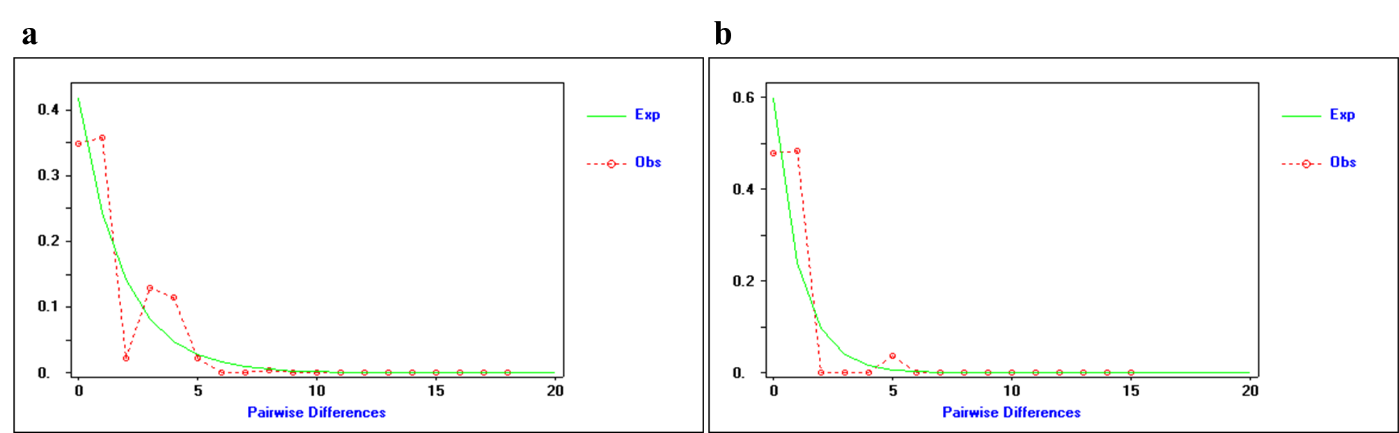

Supplement: Supplementary file 10 — Additional file 10. Supplementary data of molecular phylogeny data based on ITS2. This file contains Tables 1–5 and Figs. 1–9. [file 12936_2022_4121_MOESM10_ESM.docx]
